# A Caputo fractional-order SEIHRD model for Ebola: theoretical analysis, sensitivity, bifurcation, and numerical simulations

**DOI:** 10.1038/s41598-026-42467-2

**Published:** 2026-03-16

**Authors:** R. Malathy, G. Sai Sundara Krishnan, K. Loganathan

**Affiliations:** 1 Department of Mathematics, SNS College of Engineering, Coimbatore, Tamilnadu India; 2 Department of Applied Mathematics and Computational Sciences, PSG College of Technology, Coimbatore, Tamilnadu India; 3 Department of Mathematics and Statistics, Manipal University Jaipur, Jaipur, 303007 India

**Keywords:** SEIHRD epidemic model-Ebola virus, Caputo fractional derivative, Stability analysis, Sensitivity analysis, Bifurcation, Fractional Runge–Kutta and differential transform method, Computational biology and bioinformatics, Mathematics and computing, Physics

## Abstract

This study develops and analyzes a Caputo fractional-order SEIHRD model to investigate the transmission dynamics and control of Ebola virus disease. The model ensures positivity, boundedness, and invariance of feasible regions for all solutions. Rigorous analysis establishes local and global existence, uniqueness, and well-posedness of the system. The basic reproduction number $R_0$ is derived, with stability analysis of disease-free and endemic equilibria revealing a forward transcritical bifurcation at $R_0 = 1$. Sensitivity analysis identifies key parameters significantly influencing $R_0$ and endemic infection levels. Numerical simulations using both the Fractional Runge-Kutta scheme and the Fractional Differential Transform Method demonstrate the pronounced impact of the fractional order on system stability and persistence, with the Runge–Kutta method providing superior accuracy. These results highlight the critical role of fractional-order modeling in capturing memory effects in epidemic processes and suggest the efficacy of fractional calculus in enhancing epidemic predictions. The results show that fractional-order dynamics capture Ebola’s persistence and memory effects, providing a framework for control strategies. This also points toward incorporating fuzzy fractional approaches to better address parameter uncertainty, offering a robust framework for future extensions in epidemic modeling and control strategies.

## Introduction

Epidemic outbreaks continue to challenge global public health systems, requiring effective response and prevention strategies. Among infectious diseases, Ebola Virus Disease (EVD) demands special attention due to its high fatality rate and complex transmission mechanisms. Unlike airborne diseases, Ebola spreads primarily through direct contact with contaminated bodily fluids and through unsafe burial practices, contributing significantly to ongoing transmission chains. These features necessitate specialized models to accurately capture the multifaceted transmission routes, including post-mortem infectivity.

Mathematical models have been indispensable in understanding disease dynamics and informing control measures. Classical compartmental models, such as SEIR-type frameworks, segment populations into susceptible, exposed, infectious, and recovered groups to study epidemic progression. However, these traditional models often inadequately represent memory and hereditary properties intrinsic to biological processes, such as incubation delays and immune response dynamics.

Fractional calculus offers a powerful extension by incorporating derivatives of non-integer order, enabling the capture of long-term temporal dependencies and memory effects absent in integer-order models. This is particularly relevant for Ebola, where the dynamics of transmission, especially involving the deceased compartments, are prolonged and complex. Fractional-order models thus provide a more realistic and rich description of outbreak evolution and control.

Existing fractional SEIHRD models for Ebola remain limited in analysis and numerical treatment. Our work addresses this gap by developing a Caputo fractional-order SEIHRD model that explicitly includes deceased infectious individuals and post-mortem transmission. We conduct rigorous qualitative analyses-including positivity, boundedness, equilibria stability, and bifurcation examinations-and implement robust numerical schemes, notably the Fractional Runge-Kutta (FRK) and the Fractional Differential Transform Method (FDTM). This combined analytical and computational approach enhances model fidelity and predictive capability.

The motivation for this study lies in the critical need to incorporate memory-driven effects and transmission nuances into Ebola epidemic modeling, improving the understanding of outbreak persistence under various intervention strategies. Moreover, the research opens avenues for future integration of fuzzy fractional methods to address parameter uncertainty, further empowering epidemic forecasting and control.

Recent years have witnessed significant growth in the application of fractional calculus to epidemic modeling, as researchers leverage memory-dependent frameworks to capture long-term infectious dynamics and complex transmission pathways more accurately. For instance, fractional order models have been extensively studied in the context of Ebola virus disease, such as in “A theoretical study on fractional Ebola hemorrhagic fever model”^1^, which offers rigorous analysis and highlights the role of non-integer order dynamics in describing disease persistence.

Similar fractional approaches have been adopted for a range of other infectious diseases. Notably, “A computational study of transmission dynamics for dengue fever with a fractional approach”^2^ demonstrates how the inclusion of fractional derivatives enhances epidemic forecasting and transmission analysis. Recent investigations, such as “The analysis of a new fractional model to the Zika virus infection with mutant””^3^, further illustrate the value of this methodology for tracking emerging diseases and variants.

In addition, fractional modeling techniques have contributed to the analysis of nonlinear phenomena in mathematical physics, with works like “Multi-type solitary wave solutions of Korteweg-de-Veries (KdV) equation”^4^ providing insight into advanced solution structures. The stochastic features of disease spread are analyzed in “Numerical simulation and analysis of the stochastic HIV/AID fractional order”^5^, where randomized epidemiological behaviors are better captured using fractional frameworks.

Beyond disease modeling, fractional calculus has enabled significant advances in the study of chaotic systems and control strategies. The work “Design of a fractional-order atmospheric model via a class of ACT-like chaotic system and its sliding mode chaos control”^6^ explores the effectiveness of fractional dynamics in representing and regulating chaotic meteorological processes. Moreover, the application of fractional-order modeling to digital ecosystems is highlighted in “Chaos and stability of a fractional model of the cyber ecosystem”^7^, underscoring the versatility of these mathematical tools across disciplines. Further, review of literature is categorized below on specific area of applications.

### Foundations of fractional calculus and differential equations

The mathematical foundation of fractional calculus provides powerful tools to describe dynamical systems with memory and hereditary properties. Podlubny^8^ introduced fractional differential equations systematically in applied mathematics, while Diethelm^9^ developed a rigorous analysis of fractional differential equations using Caputo operators, with emphasis on existence, uniqueness, and numerical methods. Matignon^10^ established stability results for fractional differential equations, which remain central to control and dynamical system analysis. More recent works extended these concepts, including generalized $\psi$-Caputo derivatives and controllability results^11,12^. Dimitrov et al.^13^ proposed efficient numerical approximations of Caputo derivatives, demonstrating convergence in fractional differential equation solvers.

### Threshold parameters and epidemiological modeling

In mathematical epidemiology, the basic reproduction number $R_0$ plays a critical role in characterizing disease dynamics. Diekmann et al.^14^ formally defined $R_0$ for heterogeneous populations, while van den Driessche and Watmough ^15^ developed the next-generation matrix method for its computation. Castillo-Chavez and Song^16^ applied compartmental models to tuberculosis dynamics, illustrating threshold conditions and endemic equilibria. These results underpin the analysis of more recent epidemic models.

### Fractional-order models for Ebola virus disease

Fractional calculus has been increasingly applied to model Ebola virus dynamics. Area et al.^17^ investigated optimal control under vaccination constraints, while Singh^18^ developed a fractional Ebola model emphasizing nonlocal effects. More recent contributions extended these ideas using generalized fractional operators^19,20^. Yunus and Olayiwola^21,22^ studied fractional Caputo-Fabrizio and co-dynamic Ebola-malaria models, while Masti et al.^23^ analyzed transitions in Ebola epidemiology from a fractional perspective. Abu Hammad et al.^24^ considered incommensurate discrete fractional orders, and Yousef^25^ extended Ebola models to include transmission from animals and healthcare deficiencies. El Rhoubari et al.^26^ applied fractional-order analysis to Ebola persistence in bat populations. Farman et al.^27^ showed how fractional operators can be applied to monitor and control brain network diseases under immunotherapy. Other applications include asymptotic stability studies in Caputo sense^28^, and sensitivity-based stability results^29,30^.

### Fuzzy and hybrid fractional epidemic models

Fuzzy extensions of fractional epidemic models provide additional flexibility to capture uncertainties. Dhandapani et al.^31–33^ proposed several fuzzy fractional epidemic formulations, including death populations, retarded delay structures, and vaccination strategies. Umapathy et al.^34,35^ further developed decomposition methods and crisp/fuzzy solution comparisons. These fuzzy-fractional approaches complement classical epidemic models by integrating uncertainty and imprecision into disease dynamics.

### Numerical and control approaches

Beyond theoretical formulations, numerical and control frameworks have been developed to simulate and mitigate Ebola dynamics. Vellappandi et al.^36^ and Rosa and Ndairou^20^ applied optimal control methods to fractional epidemic systems, while Ahmed et al.^29^ provided sensitivity analysis and implications for Ebola interventions. Umapathy and collaborators^34,35^ and Dimitrov et al.^13^ emphasized computational schemes, ensuring reliable approximation of fractional operators in epidemic contexts.

### Summary of literature review

Overall, the literature highlights two converging trends: (i) the development of fractional-order frameworks for epidemic models that capture memory, nonlocality, and uncertainty; and (ii) the application of these frameworks to Ebola virus disease, leading to deeper understanding of thresholds, stability, and optimal control. The present work builds on these contributions by constructing a Caputo fractional-order SEIHRD Ebola model, analyzing equilibria and bifurcations, and validating the dynamics through numerical simulations.

### Novelty

The novelty of our work in this study is the development of a Caputo fractional-order SEIHRD model explicitly including deceased infectious individuals, rigorous qualitative analysis including bifurcation results, and the comparative application of advanced numerical techniques (FRK and FDTM) for epidemic modeling, which to our knowledge has not been comprehensively explored in the existing literature.

The above “Introduction” introduces the topic, the “Preliminaries on the Caputo fractional derivative” provides a preliminaries on Caputo derivative, the “Fractional Ebola model (Caputo)” formulates the fractional Ebola model, “Qualitative analyis for fractional SEIHRD Ebola model” presents the qualitative analysis, “Numerical analysis” describes the numerical approach, “Results and discussion” discusses simulation results, and “Conclusion and future direction” concludes with future directions.

## Preliminaries on the Caputo fractional derivative

Fractional calculus generalizes classical differentiation and integration to non-integer (fractional) orders, enabling the modeling of systems with memory and hereditary properties. Among several formulations, the Caputo fractional derivative^37^ is widely used in applications due to its compatibility with standard initial conditions.

### Definition 2.1

*(Caputo fractional derivative)* Let *f*(*x*) be a sufficiently smooth function on $x \ge 0$, and let $\alpha > 0$ be a real (non-integer) order. The Caputo fractional derivative of order $\alpha$ is defined as

1 $$\begin{aligned} ^{\textrm{C}}D_{a}^{\alpha } f(x) = \frac{1}{\Gamma (n - \alpha )} \int _{a}^{x} \frac{f^{(n)}(t)}{(x - t)^{\alpha + 1 - n}} \, dt, \quad n = \lceil \alpha \rceil , \end{aligned}$$

where $\Gamma (\cdot )$ is the Gamma function, *n* is the smallest integer greater than or equal to $\alpha$, and $f^{(n)}$ is the *n*-th derivative of *f*^37^.

Since there are many fractional derivatives we prefered and used the Caputa derivative because,

Mathematical advantages:

- The Caputo derivative preserves the original initial conditions (Cauchy problem well-posedness)
- Unlike Riemann–Liouville operators, Caputo allows incorporation of integer-order derivatives
- Computational efficiency for numerical schemes (FRK, FDTM)

Biological justification:

- Captures memory effects in disease incubation and immune response persistence
- Represents delayed effects in transmission from deceased individuals
- Accurately models long-term epidemiological dependencies

Comparative context:

- Riemann–Liouville: computational limitations at initial conditions
- Atangana–Baleanu: computational complexity without additional biological insight for this system
- Caputo–Fabrizio: less suited for epidemic memory effects

## Fractional Ebola model (Caputo)

The Ebola model with susceptible *S*, exposed *E*, infectious *I*, hospitalized *H*, recovered *R*, and Ebola-related deceased *D* is

2 $$\begin{aligned} \left\{ \begin{array}{l} {}^{C}\!D_t^\alpha S(t) = \Lambda - \lambda (t)\,S(t) - \mu S(t), \\ {}^{C}\!D_t^\alpha E(t) = \lambda (t)\,S(t) - (\sigma +\mu )E(t), \\ {}^{C}\!D_t^\alpha I(t) = \sigma E(t) - (\gamma + \delta + \mu + \kappa ) I(t), \\ {}^{C}\!D_t^\alpha H(t) = \kappa I(t) - (\gamma _h + \delta _h + \mu ) H(t), \\ {}^{C}\!D_t^\alpha R(t) = \gamma I(t) + \gamma _h H(t) - \mu R(t), \\ {}^{C}\!D_t^\alpha D(t) = \delta I(t) + \delta _h H(t) - \eta D(t), \end{array} \right. \end{aligned}$$

with force of infection

$$\begin{aligned} \lambda (t)=\beta \frac{I}{N}+\beta _h\frac{H}{N}+\beta _d\frac{D}{N},\qquad N=S+E+I+H+R+D. \end{aligned}$$

All parameters are nonnegative and $0<\alpha \le 1$ with meanings: $\beta ,\beta _h,\beta _d$ (transmission rates), $\sigma$ (progression $E\rightarrow I$), $\gamma ,\gamma _h$ (recovery), $\delta ,\delta _h$ (disease-induced death), $\kappa$ (hospitalization), $\eta$ (safe burial), $\Lambda$ (recruitment), $\mu$ (natural death). initial conditions

$$\begin{aligned} S(0)=S_0,\; E(0)=E_0,\; I(0)=I_0,\; H(0)=H_0,\; R(0)=R_0,\; D(0)=D_0, \end{aligned}$$

where $S_0,\ldots ,D_0\ge 0$. Let us present below the compartment description in Table 1, and the parameter description in Table 2.

**Table 1:** The model partitions: the population into six epidemiologically meaningful compartments.

Symbol	Description
*S*(*t*)	Total susceptible individuals at time *t*
*E*(*t*)	Total exposed (infected but not yet infectious) individuals
*I*(*t*)	Total infectious individuals in the community
*H*(*t*)	Total hospitalized infectious individuals
*R*(*t*)	Total recovered individuals
*D*(*t*)	Total deceased individuals (not yet safely buried)

**Table 2:** Parameter values for the SEIHRD Ebola model. Values cited from^17^ or assumed (in brackets) where indicated.

Parameter	Description	Value
$\Lambda$	Recruitment rate of susceptible individuals (birth or immigration)	14, 000 (assumed)
$\mu$	Natural death rate (applies to all living compartments)	$14/1000 \approx 0.014{{day}^{-1}}$ ^17^
$\beta$	Transmission rate from community infectious individuals	$0.14{{day}^{-1}}$ ^17^
$\beta _h$	Spreading rate from hospitalized individuals	$0.29{{day}^{-1}}$ ^17^
$\beta _d$	Spreading rate from deceased individuals	$0.40{{day}^{-1}}$ ^17^
$\sigma$	Rate at which exposed individuals become infectious	$1/11.4 \approx 0.0877{{day}^{-1}}$ ^17^
$\kappa$	Hospitalization rate of infectious individuals	$1/5 = 0.2{{day}^{-1}}$ ^17^
$\gamma$	Recovery rate of community infectious individuals	$0.1{{day}^{-1}}$ (assumed)
$\gamma _h$	Recovery rate of hospitalized individuals	$0.125{{day}^{-1}}$ (assumed)
$\delta$	Disease-induced deceased rate of community infectious individuals	$1/9.6 \approx 0.104{{day}^{-1}}$ ^17^
$\delta _h$	Disease-induced deceased rate of hospitalized individuals	$0.08{{day}^{-1}}$ (assumed)
$\eta$	Burial rate (removal of deceased individuals)	$0.5{{day}^{-1}}$ (assumed)
$\alpha$	Fractional order of the Caputo derivative	$0<\alpha <1$ (assumed)

### Epidemiological significance of the unsafe burials

**Fig. 1:**
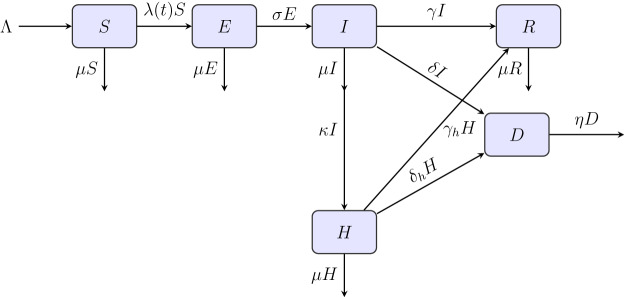
Schematic diagram of the Ebola transmission model compartments and transitions.

Unsafe burials have been documented as major transmission routes in Ebola epidemiology^17^. Post-mortem transmission accounts for significant infection chains in endemic regions. The D compartment explicitly captures this often-overlooked but critical pathway. Unlike classical SEIR models, our SEIHRD formulation distinguishes between Infectious (*I*), representing community transmission; Hospitalized (*H*), denoting controlled environments; and Deceased (*D*), representing individuals not yet safely buried who continue to contribute to transmission. The burial rate parameter $(\eta )$ allows modeling of interventions such as rapid burial protocols. This approach reflects the reality in resource-limited settings where safe burial is challenging, enables analysis of the impact of burial practices on outbreak control, and provides a quantitative framework for burial intervention policies (Fig. 1).

## Qualitative analyis for fractional SEIHRD Ebola model

In this section, all kinnds of qualitative analysis such as the positivity of the solutions analysis, invariant region analysis, boundedness, existence and uniqueness of solutions, equilibria, basic reproduction number, local and the global stability analysis of disease free and endemic equilibrium points, bifurcation analysis and the sensityvity analysis.

### Existence and uniqueness of solutions

We rewrite the SEIHRD system in compact vector form. Let

$$\begin{aligned} X(t)=\begin{pmatrix} S(t)\\ E(t)\\ I(t)\\ H(t)\\ R(t)\\ D(t)\end{pmatrix}, \qquad X_0=\begin{pmatrix} S_0\\ E_0\\ I_0\\ H_0\\ R_0\\ D_0\end{pmatrix}\in \mathbb {R}_+^6. \end{aligned}$$

The Caputo fractional-order SEIHRD model (system) may be written as

3a $$\begin{aligned} {}^{C}\!D_t^\alpha X(t) = F(X(t)), \qquad X(0)=X_0, \end{aligned}$$

where the right-hand side $F:\mathbb {R}_+^6\rightarrow \mathbb {R}^6$ has components

3b $$\begin{aligned} f_1(S,E,I,H,R,D)&=\Lambda - \lambda (S,E,I,H,R,D)\,S - \mu S, \end{aligned}$$

3c $$\begin{aligned} f_2(S,E,I,H,R,D)&=\lambda (S,E,I,H,R,D)\,S - (\sigma +\mu )E, \end{aligned}$$

3d $$\begin{aligned} f_3(S,E,I,H,R,D)&=\sigma E - (\gamma +\delta +\mu +\kappa )I, \end{aligned}$$

3e $$\begin{aligned} f_4(S,E,I,H,R,D)&=\kappa I - (\gamma _h+\delta _h+\mu )H, \end{aligned}$$

3f $$\begin{aligned} f_5(S,E,I,H,R,D)&=\gamma I + \gamma _h H - \mu R, \end{aligned}$$

3g $$\begin{aligned} f_6(S,E,I,H,R,D)&=\delta I + \delta _h H - \eta D. \end{aligned}$$

Here the force of infection is

3h $$\begin{aligned} \lambda (S,E,I,H,R,D)=\frac{\beta I + \beta _h H + \beta _d D}{N},\qquad N=S+E+I+H+R+D. \end{aligned}$$

We make the biologically natural assumption that the initial total population

4 $$\begin{aligned} N_0:= {\bf 1}^\top X_0 = S_0+E_0+I_0+H_0+R_0+D_0 > 0, \end{aligned}$$

and that all model parameters are nonnegative.

#### Theorem 4.1

(Local existence and uniqueness) *Let* $0<\alpha \le 1$ *and let* $X_0\in \mathbb {R}_+^6$ *satisfy* (4).* Then there exists* $T^*>0$ *and a unique continuous function* $X\in C([0,T^*],\mathbb {R}^6)$ *which is a (mild) solution of* (3a) *in the Volterra (integral) sense*:

5 $$\begin{aligned} X(t)=X_0+\frac{1}{\Gamma (\alpha )}\int _0^t (t-s)^{\alpha -1} F(X(s))\,ds, \qquad t\in [0,T^*]. \end{aligned}$$

*Moreover the solution is unique on* $[0,T^*]$.

#### Proof

The proof is standard but we give all details for completeness.

Step 1. (Local smoothness / Lipschitz property of *F*.) Consider a closed ball centered at $X_0$,

6 $$\begin{aligned} B_R(X_0)=\{ X\in \mathbb {R}^6:\; \Vert X-X_0\Vert _\infty \le R\}, \end{aligned}$$

with $R>0$ chosen so small that for all $X\in B_R(X_0)$ the total population $N={\bf 1}^\top X$ satisfies $N\ge m>0$ for some *m* (this is possible because $N_0>0$ and the components are continuous in *X*). On the set $B_R(X_0)$ the mapping *F* is continuously differentiable (because each $f_i$ is a rational function whose denominator *N* is bounded away from zero on $B_R(X_0)$). Hence *F* is Lipschitz on $B_R(X_0)$: there exists $L>0$ such that for all $X,Y\in B_R(X_0)$

7 $$\begin{aligned} \Vert F(X)-F(Y)\Vert _\infty \le L \Vert X-Y\Vert _\infty . \end{aligned}$$

(One may take $L=\sup _{Z\in B_R(X_0)} \Vert JF(Z)\Vert _{\infty }$ where *JF* denotes the Jacobian matrix of *F*.)

Step 2. (Volterra reformulation.) A function $X\in C([0,T],\mathbb {R}^6)$ is a solution of (3a) iff it satisfies the Volterra integral equation (5) (this is the standard equivalence for Caputo problems; see e.g.^8,9^).

Step 3. (Banach space and the integral operator). Fix $T>0$ (to be chosen). Let

$$\begin{aligned} \mathcal {X}_T:=\big (C([0,T],\mathbb {R}^6),\Vert \cdot \Vert _{T}\big ),\qquad \Vert X\Vert _T:=\sup _{t\in [0,T]}\Vert X(t)\Vert _\infty , \end{aligned}$$

which is a Banach space. Define the operator $\mathcal {T}:\mathcal {X}_T\rightarrow \mathcal {X}_T$ by

8 $$\begin{aligned} (\mathcal {T}X)(t):=X_0+\frac{1}{\Gamma (\alpha )}\int _0^t (t-s)^{\alpha -1} F(X(s))\,ds. \end{aligned}$$

We wish to apply the Banach fixed-point theorem to $\mathcal {T}$ on the closed subset

$$\begin{aligned} \mathcal {B}:=\{X\in \mathcal {X}_T:\; \Vert X-X_0\Vert _T\le R\}, \end{aligned}$$

where *R* is the same as in (6) and chosen so that *F* is Lipschitz on $B_R(X_0)$.

Step 4. ( $\mathcal {T}$ maps $\mathcal {B}$ into $\mathcal {B}$). If $X\in \mathcal {B}$ then for $t\in [0,T]$,

$$\begin{aligned} \begin{aligned} \Vert (\mathcal {T}X)(t)-X_0\Vert _\infty&\le \frac{1}{\Gamma (\alpha )}\int _0^t (t-s)^{\alpha -1}\Vert F(X(s))\Vert _\infty \,ds \\&\le \frac{M_F}{\Gamma (\alpha )}\int _0^t (t-s)^{\alpha -1}\,ds = \frac{M_F}{\Gamma (\alpha +1)} t^{\alpha }, \end{aligned} \end{aligned}$$

where $$M_F:= \sup \{ \Vert F(Z)\Vert _\infty : Z\in B_R(X_0)\}<\infty$$. Hence

9 $$\begin{aligned} \Vert \mathcal {T}X-X_0\Vert _T \le \frac{M_F}{\Gamma (\alpha +1)} T^{\alpha }. \end{aligned}$$

By choosing $T>0$ sufficiently small so that

10 $$\begin{aligned} \frac{M_F}{\Gamma (\alpha +1)} T^{\alpha } \le R, \end{aligned}$$

we ensure $$\mathcal {T}(\mathcal {B})\subset \mathcal {B}$$.

Step 5. (Contraction property). Let $X,Y\in \mathcal {B}$. Using (7) we obtain for $t\in [0,T]$,

$$\begin{aligned} \begin{aligned} \Vert (\mathcal {T}X)(t)-(\mathcal {T}Y)(t)\Vert _\infty&\le \frac{1}{\Gamma (\alpha )}\int _0^t (t-s)^{\alpha -1}\Vert F(X(s))-F(Y(s))\Vert _\infty \,ds \\&\le \frac{L}{\Gamma (\alpha )} \Vert X-Y\Vert _T \int _0^t (t-s)^{\alpha -1}\,ds \\&= \frac{L}{\Gamma (\alpha +1)} t^{\alpha }\, \Vert X-Y\Vert _T. \end{aligned} \end{aligned}$$

Taking the supremum over $t\in [0,T]$ yields

11 $$\begin{aligned} \Vert \mathcal {T}X-\mathcal {T}Y\Vert _T \le q \, \Vert X-Y\Vert _T, \qquad q:= \frac{L}{\Gamma (\alpha +1)} T^{\alpha }. \end{aligned}$$

Choose $T>0$ (possibly smaller than above) so that $q<1$. Then $\mathcal {T}$ is a contraction on $\mathcal {B}$.

Step 6. (Utilising Banach fixed-point theorem). With $T>0$ satisfying (10) and $q<1$ from (11), the operator $\mathcal {T}$ has a unique fixed point $X\in \mathcal {B}$. This fixed point is the unique continuous solution of (5) on [0, *T*]. Taking $T^*$ to be such a *T* proves local existence and uniqueness on $[0,T^*]$.

Step 7. (Uniqueness on the maximal interval). The uniqueness argument above is local: if *X* and *Y* are two solutions on an interval $[0,\tau ]$, one can choose a subdivision of $[0,\tau ]$ into subintervals of length $\le T^*$ and apply the contraction argument on each subinterval to deduce $X\equiv Y$ on $[0,\tau ]$. Hence the solution is unique on its maximal interval of existence. $\square$

#### Remark 4.1

*(Extension to a maximal interval and global existence)* Let $[0,T_{\max })$ be the maximal interval of existence of the unique solution constructed above. Standard continuation theory for Caputo problems (by repeated application of the local existence result) implies either $T_{\max }=\infty$ or $\limsup _{t\rightarrow T_{\max }^-}\Vert X(t)\Vert _\infty = \infty$. Consequently, if one knows a priori that the solution remains bounded for all $t\ge 0$ (for example by the Invariant Region / Boundedness Theorem proved earlier which gives $N(t)\le N_{\max }$ and nonnegativity), then the blow-up alternative cannot occur and the solution extends to $[0,\infty )$. Thus, combining Theorems 4.1 and 4.3 yields global existence and uniqueness of the solution for all $t\ge 0$.

### Positivity of solutions

We consider the fractional SEIHRD model (Caputo derivative) introduced in  “Fractional Ebola model (Caputo)”:

#### Theorem 4.2

(Positivity) *If the initial data satisfy* $S_0,E_0,I_0,H_0,R_0,D_0\ge 0$ *and all parameters are nonnegative, then the solution*

$$\begin{aligned} (S(t),E(t),I(t),H(t),R(t),D(t)) \end{aligned}$$

*of the initial value problem exists (at least on a maximal interval of existence) and satisfies*

$$\begin{aligned} S(t),E(t),I(t),H(t),R(t),D(t)\ge 0\quad \text {for all } t\ge 0. \end{aligned}$$

#### Proof

We give a classical proof based on the equivalent Volterra integral form for Caputo problems and a first-crossing-time contradiction.

Step 1: Equivalent integral formulation. For a scalar function $x(t)$ and $0<\alpha \le 1$, the Caputo initial-value problem

$$\begin{aligned} {}^{C}\!D_t^\alpha x(t)=f(t,x(t)),\qquad x(0)=x_0, \end{aligned}$$

is equivalent to the Volterra integral equation

$$\begin{aligned} x(t)=x_0+\frac{1}{\Gamma (\alpha )}\int _0^t (t-s)^{\alpha -1} f(s,x(s))\,ds, \end{aligned}$$

whenever $f$ is continuous and a (mild) solution exists. Apply this componentwise to our system to obtain, for example,

$$\begin{aligned} S(t)=S_0+\frac{1}{\Gamma (\alpha )}\int _0^t (t-s)^{\alpha -1}\big (\Lambda - \lambda (s)S(s)-\mu S(s)\big )\,ds, \end{aligned}$$

and analogous integral formulae for $E,I,H,R,D$.

Step 2: Nonnegativity of the right-hand side at zero. Observe that for nonnegative state vectors,

- at $$S=0$$: $$\Lambda - \lambda S -\mu S = \Lambda \ge 0$$,
- at $$E=0$$: $$\lambda S -(\sigma +\mu )E = \lambda S \ge 0$$,
- at $$I=0$$: $$\sigma E -(\gamma +\delta +\mu +\kappa )I = \sigma E \ge 0$$,
- at $$H=0$$: $$\kappa I -(\gamma _h+\delta _h+\mu )H = \kappa I \ge 0$$,
- at $$R=0$$: $$\gamma I + \gamma _h H -\mu R = \gamma I + \gamma _h H \ge 0$$,
- at $$D=0$$: $$\delta I + \delta _h H -\eta D = \delta I + \delta _h H \ge 0$$.

Hence whenever a compartment variable vanishes while the others are nonnegative, the corresponding right-hand side is nonnegative.

Step 3: Contradiction by first-crossing time. Assume, to the contrary, that some component becomes negative at some time. Since the solution components are continuous (by existence theory for Caputo systems under standard Lipschitz/continuity hypotheses), there exists a first time $t^*>0$ and an index $k\in \{S,E,I,H,R,D\}$ such that

$$\begin{aligned} x_k(t)\ge 0\ \text {for } 0\le t\le t^*,\qquad x_k(t^*)=0, \end{aligned}$$

and $x_k(t)<0$ for $t>t^*$ in a right neighborhood of $t^*$. (Here $x_k$ denotes the $k$-th compartment function.) Also, at $t^*$ all other components satisfy $x_j(t^*)\ge 0$ because $t^*$ is the first time any component hits zero and then becomes negative.

Take the integral formulation for $x_k$:

$$\begin{aligned} x_k(t)=x_k(0)+\frac{1}{\Gamma (\alpha )}\int _0^t (t-s)^{\alpha -1} f_k(s,X(s))\,ds, \end{aligned}$$

where $f_k$ is the right-hand side function of the $k$-th equation (continuous in $s$ and the state). Evaluate this at $t=t^*$:

$$\begin{aligned} x_k(t^*)=x_k(0)+\frac{1}{\Gamma (\alpha )}\int _0^{t^*} (t^*-s)^{\alpha -1} f_k(s,X(s))\,ds. \end{aligned}$$

By the choice of $t^*$, for all $s\in [0,t^*]$ the state components are nonnegative, so from Step 2 we have $f_k(s,X(s))\ge 0$ for all $s\in [0,t^*]$. The kernel $(t^*-s)^{\alpha -1}$ is nonnegative on $[0,t^*]$, and $\Gamma (\alpha )>0$. Therefore the integral term is nonnegative, and since $x_k(0)\ge 0$, it follows that

$$\begin{aligned} x_k(t^*)\ge 0. \end{aligned}$$

But by definition $x_k(t^*)=0$. Thus the integral term must be zero:

$$\begin{aligned} \int _0^{t^*} (t^*-s)^{\alpha -1} f_k(s,X(s))\,ds = 0. \end{aligned}$$

Because the integrand is continuous and nonnegative on $[0,t^*]$, the integral being zero implies $f_k(s,X(s))=0$ for all $s\in [0,t^*]$. In particular, in a right neighborhood of $t^*$ we still have $f_k(s,X(s))\ge 0$. Returning to the integral representation for times $t>t^*$ close to $t^*$, we get

$$\begin{aligned} x_k(t)=\frac{1}{\Gamma (\alpha )}\int _0^{t} (t-s)^{\alpha -1} f_k(s,X(s))\,ds \ge 0, \end{aligned}$$

for $t$ near $t^*$. This contradicts the assumption that $x_k(t)<0$ for $t>t^*$ sufficiently close to $t^*$. Hence no component can become negative.

Step 4: Conclusion. Since the choice of $k$ was arbitrary, all components remain nonnegative for all $t$ in the interval of existence. This proves the claimed positivity property. $\square$

### Invariant region and boundedness

Recall the fractional SEIHRD system from “Fractional Ebola model (Caputo)” and let

12 $$\begin{aligned} M(t)=S(t)+E(t)+I(t)+H(t)+R(t), \qquad N(t)=M(t)+D(t). \end{aligned}$$

#### Theorem 4.3

(Invariant region / boundedness) *Assume nonnegative initial data* $S_0,E_0,I_0,H_0,R_0,D_0\ge 0$ *and nonnegative parameters* $\Lambda ,\mu ,\sigma ,\gamma ,\gamma _h,\delta ,\delta _h,\kappa ,\eta ,\beta ,\beta _h,\beta _d$. *Then the solutions of the fractional SEIHRD model satisfy*

$$\begin{aligned} 0 \le S(t),E(t),I(t),H(t),R(t),D(t) \le N_{\max }\qquad \text {for all } t\ge 0, \end{aligned}$$

*where one may choose the finite constant*

13 $$\begin{aligned} N_{\max } \;=\; \underbrace{\max \{M(0),\Lambda /\mu \}}_{=:Y_{\max }} \;+\;\max \Big \{D(0),\,\tfrac{{\bar{\delta }}\,Y_{\max }}{\eta }\Big \}, \qquad {\bar{\delta }}=\max \{\delta ,\delta _h\}. \end{aligned}$$

*In particular the set*

$$\begin{aligned} \Omega =\{(S,E,I,H,R,D)\in \mathbb {R}_+^6:\;0\le N\le N_{\max }\} \end{aligned}$$

*is positively invariant*.

#### Proof

Step 1—Bound for $M(t)$. Summing the first five equations (living compartments) gives

14 $$\begin{aligned} {}^{C}\!D_t^\alpha M(t) = \Lambda -\mu M(t) - (\delta I+\delta _h H). \end{aligned}$$

Since $\delta I+\delta _h H\ge 0$, we obtain the inequality

15 $$\begin{aligned} {}^{C}\!D_t^\alpha M(t) \le \Lambda - \mu M(t). \end{aligned}$$

The scalar comparison system

16 $$\begin{aligned} {}^{C}\!D_t^\alpha y(t) = \Lambda - \mu y(t), \qquad y(0)=M(0), \end{aligned}$$

has the explicit solution

$$\begin{aligned} y(t)=\frac{\Lambda }{\mu } + \big (M(0)-\tfrac{\Lambda }{\mu }\big ) E_\alpha (-\mu t^\alpha ), \end{aligned}$$

with $E_\alpha$ the Mittag–Leffler function. As $0<E_\alpha (-\mu t^\alpha )\le 1$, we deduce

$$\begin{aligned} y(t)\le \max \{M(0),\Lambda /\mu \} =: Y_{\max }. \end{aligned}$$

From the comparison principle applied to (15), we obtain

17 $$\begin{aligned} M(t)\le Y_{\max }, \qquad t\ge 0. \end{aligned}$$

Step 2—Bound for $D(t)$. The deceased compartment satisfies

18 $$\begin{aligned} {}^{C}\!D_t^\alpha D(t) = \delta I(t) + \delta _h H(t) - \eta D(t). \end{aligned}$$

Using (17), and defining ${\bar{\delta }}=\max \{\delta ,\delta _h\}$, we have

$$\begin{aligned} \delta I(t)+\delta _h H(t) \le {\bar{\delta }}\, M(t) \le {\bar{\delta }}\, Y_{\max }. \end{aligned}$$

Thus

19 $$\begin{aligned} {}^{C}\!D_t^\alpha D(t) \le {\bar{\delta }}\, Y_{\max } - \eta D(t). \end{aligned}$$

The comparison system

20 $$\begin{aligned} {}^{C}\!D_t^\alpha w(t) = {\bar{\delta }}\, Y_{\max } - \eta w(t), \qquad w(0)=D(0), \end{aligned}$$

has solution

$$\begin{aligned} w(t)=\frac{{\bar{\delta }}\,Y_{\max }}{\eta } + \Big (D(0)-\tfrac{{\bar{\delta }}\,Y_{\max }}{\eta }\Big ) E_\alpha (-\eta t^\alpha ), \end{aligned}$$

which implies

$$\begin{aligned} w(t)\le \max \Big \{D(0),\,\tfrac{{\bar{\delta }}\,Y_{\max }}{\eta }\Big \} =: D_{\max }. \end{aligned}$$

By the comparison principle applied to (19), we obtain

21 $$\begin{aligned} D(t)\le D_{\max }, \qquad t\ge 0. \end{aligned}$$

Step 3—Bound for $N(t)$. From (17) and (21) we conclude

$$\begin{aligned} N(t)=M(t)+D(t)\le Y_{\max }+D_{\max }=N_{\max }, \end{aligned}$$

where $N_{\max }$ is defined in (13). Together with positivity of solutions (Theorem 4.2), this proves that $\Omega$ is positively invariant. $\square$

#### Theorem 4.4

(Global well-posedness) *Let* $0<\alpha \le 1$ *and let the initial condition* $X_0\in \mathbb {R}_+^6$ *satisfy* $N_0>0$ in (4). *Then the fractional SEIHRD system* (3a) *admits a unique global solution*

$$\begin{aligned} X(t)=(S(t),E(t),I(t),H(t),R(t),D(t))^\top \in \mathbb {R}_+^6, \qquad t\ge 0, \end{aligned}$$

*which enjoys the following properties*:

(i) Positivity: $X(t)\in \mathbb {R}_+^6$ for all $t\ge 0$ (Theorem 4.2).
(ii) Boundedness: The solution remains in the invariant region $\Omega$ defined in Theorem 4.3, in particular $N(t)\le N_{\max }$ for all $t\ge 0$.
(iii) Global existence and uniqueness: The solution extends uniquely to the whole half-line $[0,\infty )$.
(iv) Continuous dependence on initial data: If $X^{(1)}$ and $X^{(2)}$ are solutions corresponding to initial data $X_0^{(1)}, X_0^{(2)}\in \mathbb {R}_+^6$, then for each finite $T>0$
  $$\begin{aligned} \sup _{t\in [0,T]} \Vert X^{(1)}(t)-X^{(2)}(t)\Vert _\infty \;\le \; C_T \, \Vert X_0^{(1)}-X_0^{(2)}\Vert _\infty , \end{aligned}$$
  for some constant $C_T>0$ depending on *T* and the Lipschitz constant of *F* on the invariant region $\Omega$.

#### Proof

Items (i) and (ii) are given by Theorems 4.2 and 4.3. Item (iii) follows from Theorem 4.1 together with the continuation principle: the only obstruction to global extension would be blow-up of the solution norm, which cannot occur because of the uniform bound $N(t)\le N_{\max }$. Finally, item (iv) follows directly from the contraction argument in the proof of Theorem 4.1, which provides a Lipschitz estimate of the solution map with respect to the initial condition on any finite time interval [0, *T*]. $\square$

### Equilibria and the basic reproduction number ${R_0}$

We compute the disease-free equilibrium (DFE) and derive the basic reproduction number $R_0$ for the fractional SEIHRD model (3a) using the next-generation matrix approach^15^.

#### Disease-free-equilibrium evaluation

As there is no infection at DFE, so $E^0=I^0=H^0=D^0=0$. From the $S$-equation at equilibrium we obtain

22 $$\begin{aligned} S^0=\frac{\Lambda }{\mu }, \qquad X^0=\Big (S^0,0,0,0,0,0\Big ). \end{aligned}$$

#### Next-generation matrices

We order the infection-related compartments as

23 $$\begin{aligned} {\bf x}=(E,I,H,D)^\top , \end{aligned}$$

and the right-hand side of the system is divided as $\dot{{\bf x}} = \mathcal {F}({\bf x}) - \mathcal {V}({\bf x})$, where $\mathcal {F}$ contains new infections and $\mathcal {V}$ contains other transfers (progression, recovery, death, removal).

From (3) and (3h) the new-infection vector (only the equation for $E$ receives new infections) is

24 $$\begin{aligned} \mathcal {F}({\bf x}) = \begin{pmatrix} \lambda (S,E,I,H,R,D)\,S\\ 0\\ 0\\ 0 \end{pmatrix}. \end{aligned}$$

The Jacobian of $\mathcal {F}$ on evaluation with respect to ${\bf x}$ at the DFE $\,X^0$ (use $S^0=N^0$ at DFE) gives the matrix

25 $$\begin{aligned} F \;=\; D_{{\bf x}}\mathcal {F}\big |_{X^0} \;=\; \begin{pmatrix} 0 & \beta & \beta _h & \beta _d\\ 0 & 0 & 0 & 0\\ 0 & 0 & 0 & 0\\ 0 & 0 & 0 & 0 \end{pmatrix}, \end{aligned}$$

since $\mathcal {F}_1 = (\beta I+\beta _h H+\beta _d D)$ at the DFE.

The transfer vector $\mathcal {V}({\bf x})$ (so that $\dot{{\bf x}}=\mathcal {F}-\mathcal {V}$) is obtained from the remaining terms:

26 $$\begin{aligned} \mathcal {V}({\bf x}) = \begin{pmatrix} (\sigma +\mu )E\\ -(\sigma E) + (\gamma +\delta +\mu +\kappa )I\\ - \kappa I + (\gamma _h+\delta _h+\mu )H\\ - \delta I - \delta _h H + \eta D \end{pmatrix}. \end{aligned}$$

The Jacobian $V = D_{{\bf x}}\mathcal {V}\big |_{X^0}$ is therefore

27 $$\begin{aligned} V \;=\; \begin{pmatrix} \sigma +\mu & 0 & 0 & 0\\ -\sigma & \gamma +\delta +\mu +\kappa & 0 & 0\\ 0 & -\kappa & \gamma _h+\delta _h+\mu & 0\\ 0 & -\delta & -\delta _h & \eta \end{pmatrix}. \end{aligned}$$

Note that $V$ is nonsingular for positive parameter values and hence $V^{-1}$ exists.

#### Next-generation matrix and $R_0$

The next-generation matrix^15^ is

28 $$\begin{aligned} K \;=\; F V^{-1}. \end{aligned}$$

Because $F$ has nonzero entries only in its first row, $K$ has nonzero entries only in its first row and thus has rank at most one. Consequently $K$ has three zero eigenvalues and a single possibly nonzero eigenvalue; the basic reproduction number $R_0$ is the spectral radius of $K$, i.e.

29 $$\begin{aligned} R_0 \;=\; \rho (K) \;=\; \text {the nonzero eigenvalue of }K. \end{aligned}$$

To obtain an explicit closed form for $R_0$ we interpret infection pathways and compute their contributions (this is equivalent to evaluating the nonzero eigenvalue of $F V^{-1}$ algebraically). An individual in the exposed class $E$ progresses to $I$ with probability

30 $$\begin{aligned} p_{E\rightarrow I} \;=\; \frac{\sigma }{\sigma +\mu }, \end{aligned}$$

accounting for natural death while exposed. Once in the infectious class $I$ the average time spent (mean residence time) is

31 $$\begin{aligned} \tau _I \;=\; \frac{1}{\gamma +\delta +\mu +\kappa }. \end{aligned}$$

Thus the direct contribution to new infections from the community infectious class $I$ (per newly infected exposed individual) is

32 $$\begin{aligned} \mathcal {R}_I \;=\; p_{E\rightarrow I}\,\beta \,\tau _I \;=\; \frac{\sigma }{\sigma +\mu }\cdot \frac{\beta }{\gamma +\delta +\mu +\kappa }. \end{aligned}$$

From $I$ an individual may be hospitalized at rate $\kappa$; the fraction entering $H$ is

33 $$\begin{aligned} p_{I\rightarrow H} \;=\; \frac{\kappa }{\gamma +\delta +\mu +\kappa }. \end{aligned}$$

The mean residence time in $H$ is

34 $$\begin{aligned} \tau _H \;=\; \frac{1}{\gamma _h+\delta _h+\mu }, \end{aligned}$$

so the contribution from the hospitalized class $H$ is

35 $$\begin{aligned} \mathcal {R}_H \;=\; p_{E\rightarrow I}\,p_{I\rightarrow H}\,\beta _h\,\tau _H \;=\; \frac{\sigma }{\sigma +\mu }\cdot \frac{\kappa }{\gamma +\delta +\mu +\kappa }\cdot \frac{\beta _h}{\gamma _h+\delta _h+\mu }. \end{aligned}$$

Finally, deceased individuals in $D$ are infectious until burial. Deceased arise from two pathways:

- direct disease-induced death in $I$ at rate $\delta$ (fraction $\delta /(\gamma +\delta +\mu +\kappa )$ of those in $I$);
- disease-induced death in $H$ at rate $\delta _h$ after being hospitalized (fraction $p_{I\rightarrow H}\cdot \delta _h/(\gamma _h+\delta _h+\mu )$ of those who reach $I$).

Thus the expected number of deceased individuals produced per exposed that reach $I$ is

36 $$\begin{aligned} \mathcal {D} \;=\; \frac{\sigma }{\sigma +\mu }\cdot \frac{1}{\gamma +\delta +\mu +\kappa }\Bigg (\delta \;+\; \frac{\kappa \,\delta _h}{\gamma _h+\delta _h+\mu }\Bigg ). \end{aligned}$$

Each deceased individual remains infectious on average $\tau _D=1/\eta$ and transmits at rate $\beta _d$; hence the contribution from deceased individuals is

37 $$\begin{aligned} \mathcal {R}_D \;=\; \beta _d\,\tau _D\,\mathcal {D} \;=\; \frac{\beta _d}{\eta }\cdot \frac{\sigma }{\sigma +\mu }\cdot \frac{1}{\gamma +\delta +\mu +\kappa }\Bigg (\delta \;+\; \frac{\kappa \,\delta _h}{\gamma _h+\delta _h+\mu }\Bigg ). \end{aligned}$$

Summing the contributions $\mathcal {R}_I,\mathcal {R}_H,\mathcal {R}_D$ yields the explicit expression for $R_0$:

38 $$\begin{aligned} \boxed {\; \begin{aligned} R_0&= \mathcal {R}_I + \mathcal {R}_H + \mathcal {R}_D \\&= \frac{\sigma }{\sigma +\mu }\cdot \frac{1}{\gamma +\delta +\mu +\kappa }\Bigg ( \beta \;+\; \frac{\kappa \,\beta _h}{\gamma _h+\delta _h+\mu } \;+\; \frac{\beta _d}{\eta }\Big (\delta +\frac{\kappa \,\delta _h}{\gamma _h+\delta _h+\mu }\Big ) \Bigg ). \end{aligned} \;} \end{aligned}$$

##### Remark 4.2

The expression (38) is algebraically equal to the spectral radius $\rho (FV^{-1})$ obtained from (28). The pathway decomposition above gives clear biological interpretation: the prefactor $\dfrac{\sigma }{\sigma +\mu }$ accounts for progression from exposed to infectious (surviving natural death), the factor $\dfrac{1}{\gamma +\delta +\mu +\kappa }$ is the mean time in the community infectious state, and the bracketed sum aggregates contributions from the community infectious class $I$, hospitalized class $H$ (entered via $\kappa$), and deceased class $D$ (accumulating deaths from $I$ and $H$).

#### Numerical evaluation of $\boldsymbol{R_0}$

Using the parameter values

$$\begin{aligned} \begin{aligned}&\beta =0.14,\quad \beta _h=0.29,\quad \beta _d=0.40,\\&\sigma =\tfrac{1}{11.4}\approx 0.0877192983,\quad \kappa =0.2,\\&\gamma =0.1,\quad \gamma _h=0.125,\quad \delta =\tfrac{1}{9.6}\approx 0.1041666667,\\&\delta _h=0.08,\quad \eta =0.5,\quad \mu =0.014, \end{aligned} \end{aligned}$$

we evaluate each factor in $(38)$.

The progression prefactor

39 $$\begin{aligned} \frac{\sigma }{\sigma +\mu } = \frac{0.0877192983}{0.0877192983 + 0.014} \approx 0.8623663332. \end{aligned}$$

The mean residence time in the community infectious class

40 $$\begin{aligned} \frac{1}{\gamma +\delta +\mu +\kappa } = \frac{1}{0.1 + 0.1041666667 + 0.014 + 0.2} \approx 2.3913909924. \end{aligned}$$

The bracketed transmission contributions:

41 $$\begin{aligned} \text {community (}I\text {):}\quad&\beta = 0.14, \end{aligned}$$

42 $$\begin{aligned} \text {hospital (}H\text {):}\quad&\frac{\kappa \,\beta _h}{\gamma _h+\delta _h+\mu } = \frac{0.2\times 0.29}{0.125 + 0.08 + 0.014} \approx 0.2648401826, \end{aligned}$$

43 $$\begin{aligned} \text {deceased (}D\text {):} \nonumber \\ \quad&\frac{\beta _d}{\eta }\Big (\delta +\frac{\kappa \,\delta _h}{\gamma _h+\delta _h+\mu }\Big ) = \frac{0.40}{0.5}\Big (0.1041666667 + \frac{0.2\times 0.08}{0.125+0.08+0.014}\Big ) \nonumber \\ &\approx 0.1417808219. \end{aligned}$$

Summing the bracketed terms gives

44 $$\begin{aligned} \beta + \frac{\kappa \beta _h}{\gamma _h+\delta _h+\mu } + \frac{\beta _d}{\eta }\Big (\delta +\frac{\kappa \delta _h}{\gamma _h+\delta _h+\mu }\Big ) \approx 0.14 + 0.2648401826 + 0.1417808219 \approx 0.5466210045. \end{aligned}$$

Finally, combining $(39)$, $(40)$ and $(44)$,

45 $$\begin{aligned} \begin{aligned} R_0&= \frac{\sigma }{\sigma +\mu }\cdot \frac{1}{\gamma +\delta +\mu +\kappa } \Bigg (\beta + \frac{\kappa \beta _h}{\gamma _h+\delta _h+\mu } + \frac{\beta _d}{\eta }\Big (\delta +\frac{\kappa \delta _h}{\gamma _h+\delta _h+\mu }\Big )\Bigg )\\&\approx 0.8623663332 \times 2.3913909924 \times 0.5466210045\\&\approx \boxed {1.1272719443}. \end{aligned} \end{aligned}$$

### Local stability of the disease-free equilibrium

#### Theorem 4.5

(Local stability of the DFE) *Let* $X^0$ *be the disease-free equilibrium given in* (22) *and let* $R_0$ *be the basic reproduction number defined in* (38). *For the Caputo fractional-order SEIHRD system with* $0<\alpha \le 1$ *the DFE* $X^0$ *is locally asymptotically stable if* $R_0<1$ *and unstable if* $R_0>1$.

#### Proof

Evaluate the Jacobian *J*(*X*) of the full system at the DFE $X^0$; denote by $J_{inf}$ the $4\times 4$ submatrix that corresponds to the infection compartments ${\bf x}=(E,I,H,D)^\top$ (see (27) and (25)). One can write the linearization in block form so that the characteristic equation factorizes into terms associated with noninfectious variables (whose eigenvalues are strictly negative: $-\mu ,-\mu ,-\eta$, etc.) and the infection block determined by

46 $$\begin{aligned} {}^{C}\!D_t^\alpha {\bf x}(t) = \big (F - V\big )\,{\bf x}(t), \end{aligned}$$

where *F* and *V* are the next-generation matrices defined in (25) and (27).

Introduce the next-generation matrix

47 $$\begin{aligned} K \;=\; F V^{-1}. \end{aligned}$$

By construction $K$ is nonnegative and $R_0=\rho (K)$ is its spectral radius. The eigenvalues $\lambda$ of the linear infection operator $F-V$ are related to the eigenvalues $\mu$ of $K$ via the relation

48 $$\begin{aligned} \det (F-V-\lambda I)=0 \quad \Longleftrightarrow \quad \det \big (V(\,K - (1+\lambda V^{-1})\,)\big )=0, \end{aligned}$$

and in particular the sign of the real parts of eigenvalues of $F-V$ is governed by whether $\rho (K)$ is less than, equal to, or greater than $1$. More concretely, standard next-generation matrix theory (see^14,15^) gives that if $R_0<1$ then all eigenvalues of $F-V$ have strictly negative real parts, whereas if $R_0>1$ then $F-V$ possesses an eigenvalue with positive real part.

For fractional systems with Caputo derivative of order $0<\alpha \le 1$, local asymptotic stability of the equilibrium is characterized by Matignon’s condition: an equilibrium is locally asymptotically stable if every eigenvalue $\lambda$ of the linearization satisfies

49 $$\begin{aligned} |\arg (\lambda )| > \frac{\alpha \pi }{2}. \end{aligned}$$

In particular, if all eigenvalues have strictly negative real parts (i.e. their arguments are $\pi$), then (49) is satisfied for every $0<\alpha \le 1$. Therefore, when $R_0<1$ we obtain that all eigenvalues of the Jacobian at $X^0$ satisfy (49) and the DFE is locally asymptotically stable. Conversely, if $R_0>1$ an eigenvalue of $F-V$ has positive real part and the Matignon condition is violated, so $X^0$ is unstable. $\square$

### Global stability of the disease-free equilibrium

#### Theorem 4.6

(Global asymptotic stability of the DFE) *Assume the SEIHRD model satisfies the hypotheses of Theorems* 4.2–4.3 (*positivity and boundedness*) *and let* $K=FV^{-1}$ *be as in* (47). *Suppose* $K$ *admits a positive left eigenvector* $v^\top >0$ *associated with the principal eigenvalue* $R_0$, *i.e*.

50 $$\begin{aligned} v^\top K = R_0\, v^\top . \end{aligned}$$

*If* $R_0 \le 1$ *then the disease-free equilibrium* $X^0$ *is globally asymptotically stable in the feasible region* $\Omega$: *every solution of the fractional SEIHRD system with nonnegative initial data converges to* $X^0$ *as* $t\rightarrow \infty$.

#### Proof

Let ${\bf x}(t)=(E,I,H,D)^\top$ denote the infection subvector and recall the splitting $\dot{{\bf x}}=\mathcal {F}({\bf x})-\mathcal {V}({\bf x})$. For the linearized infection dynamics we may write

51 $$\begin{aligned} {}^{C}\!D_t^\alpha {\bf x}(t) = (F - V)\,{\bf x}(t) + {\bf G}({\bf x}(t)), \end{aligned}$$

where ${\bf G}({\bf x})$ collects higher-order terms arising from the nonlinear dependence of the force of infection on the full state; note that ${\bf G}({\bf x})$ satisfies ${\bf G}({\bf x})\le 0$ componentwise for ${\bf x}\ge 0$ because the true force of infection is $\lambda (t)=\dfrac{\beta I+\beta _h H+\beta _d D}{N}\le \dfrac{\beta I+\beta _h H+\beta _d D}{N^0}$ for $N(t)\ge 0$ and $N(t)\le N_{\max }$ (boundedness). The inequality sign is used in an entrywise sense; this is the standard monotonicity used in the comparison principle for compartmental models.

Choose the positive row vector $v^\top >0$ satisfying (50). Define the linear Lyapunov function

52 $$\begin{aligned} V(t):= v^\top {\bf x}(t) = v_1 E(t) + v_2 I(t) + v_3 H(t) + v_4 D(t), \end{aligned}$$

which is nonnegative and vanishes if and only if ${\bf x}={\bf 0}$. Compute its Caputo derivative along solutions using (51):

53 $$\begin{aligned} {}^{C}\!D_t^\alpha V(t) = v^\top {}^{C}\!D_t^\alpha {\bf x}(t) = v^\top (F - V){\bf x}(t) + v^\top {\bf G}({\bf x}(t)). \end{aligned}$$

Using (50) we have $v^\top F = R_0\, v^\top V$. Thus

54 $$\begin{aligned} v^\top (F - V){\bf x} = v^\top V (R_0 - 1){\bf x}. \end{aligned}$$

Since $V$ is a nonsingular M-matrix (diagonal dominant with positive diagonal), the row vector $v^\top V$ is nonnegative and strictly positive unless ${\bf x}=0$. Hence there exists a constant $c>0$ (depending only on $v$ and $V$) such that

55 $$\begin{aligned} v^\top V {\bf x} \ge c\, \Vert {\bf x}\Vert _1 \qquad \text {for all }{\bf x}\ge 0. \end{aligned}$$

Combining (53)–(55) and using that $v^\top {\bf G}({\bf x})\le 0$ for ${\bf x}\ge 0$, we obtain the key estimate

56 $$\begin{aligned} {}^{C}\!D_t^\alpha V(t) \le (R_0 - 1)\, v^\top V {\bf x}(t) \le (R_0 - 1)\, c\, \Vert {\bf x}(t)\Vert _1. \end{aligned}$$

Now consider two cases:

Case 1: $R_0<1$. Then the right-hand side of (56) is strictly negative for any ${\bf x}(t)\ne 0$. Consequently ${}^{C}\!D_t^\alpha V(t)<0$ whenever $V(t)>0$. By the fractional Lyapunov direct method (see for instance^9,38^) this implies that $V(t)\rightarrow 0$ as $t\rightarrow \infty$, and hence ${\bf x}(t)\rightarrow {\bf 0}$. Using boundedness of the total population and the *S*-equation, one obtains $S(t)\rightarrow S^0=\Lambda /\mu$ and therefore the full state $X(t)\rightarrow X^0$ as $t\rightarrow \infty$. Thus the DFE is globally asymptotically stable in $\Omega$.

Case 2: $R_0=1$. Then (56) implies ${}^{C}\!D_t^\alpha V(t)\le 0$. From the comparison principle and LaSalle-type arguments adapted to fractional systems (see^38^) one obtains that ${\bf x}(t)$ approaches the largest invariant set contained in $\{ {\bf x}\ge 0:\ v^\top V {\bf x}=0\}$, which, by positivity of $v^\top V$, is $\{{\bf 0}\}$. Hence ${\bf x}(t)\rightarrow {\bf 0}$ and the DFE is globally attractive. With the boundedness argument as before, $X(t)\rightarrow X^0$.

Therefore for $R_0\le 1$ the DFE is globally asymptotically stable in the feasible region $\Omega$, completing the proof. $\square$

#### Remark 4.3

The existence of a strictly positive left eigenvector $v^\top >0$ for $K$ follows from the Perron–Frobenius theorem when $K$ is nonnegative and irreducible. If $K$ is reducible one may restrict attention to the irreducible block(s) that receive infection from initial conditions; the same Lyapunov construction applies to the communicating class that contains the infection. This reduction is standard in next-generation matrix analyses.

### Existence and local stability of the endemic equilibrium

#### Theorem 4.7

(Existence of an endemic equilibrium) *Assume the model parameters are nonnegative and satisfy the hypotheses of* *Theorems* 4.2*and* 4.3. *Let* $R_0$ *be the basic reproduction number given in* (38). *If* $R_0>1$, *then the fractional SEIHRD system* (3a) *admits at least one endemic equilibrium*

$$\begin{aligned} X^*=(S^*,E^*,I^*,H^*,R^*,D^*), \end{aligned}$$

with $I^*>0$ and all components nonnegative. Moreover every component of $X^*$ can be expressed algebraically in terms of $I^*>0$ by the relations

57a $$\begin{aligned} E^*&= \frac{c_I}{\sigma }\, I^*, \qquad c_I:=\gamma +\delta +\mu +\kappa , \end{aligned}$$

57b $$\begin{aligned} H^*&= \frac{\kappa }{\gamma _h+\delta _h+\mu }\, I^*, \end{aligned}$$

57c $$\begin{aligned} R^*&= \frac{\gamma I^* + \gamma _h H^*}{\mu }, \end{aligned}$$

57d $$\begin{aligned} D^*&= \frac{\delta I^* + \delta _h H^*}{\eta }, \end{aligned}$$

57e $$\begin{aligned} S^*&= \frac{\Lambda }{\mu + \lambda ^*}, \qquad \lambda ^* \;=\; \frac{\beta I^* + \beta _h H^* + \beta _d D^*}{N^*}, \end{aligned}$$

where $N^*=S^*+E^*+I^*+H^*+R^*+D^*$. Consequently the endemic equilibrium is determined by any positive root $I^*>0$ of the scalar consistency equation obtained by substituting (57) into (57e).

#### Proof

We prove existence by reducing the equilibrium equations to a single scalar consistency equation for $I^*$ and applying continuity / intermediate value arguments.

Step 1 (algebraic reduction). At equilibrium, set the Caputo derivatives to zero in (3a). From the third, fourth, fifth and sixth equations we obtain

$$\begin{aligned} \sigma E^* = c_I I^*, \qquad \kappa I^* = c_H H^*, \qquad \mu R^* = \gamma I^* + \gamma _h H^*, \qquad \eta D^* = \delta I^* + \delta _h H^*, \end{aligned}$$

where $c_I=\gamma +\delta +\mu +\kappa$ and $c_H=\gamma _h+\delta _h+\mu$. Solving these equalities yields the explicit linear relations (57a)–(57d). These relations are algebraic and valid for any $I^*\ge 0$.

The susceptible equation at equilibrium reads

$$\begin{aligned} 0 = \Lambda - \lambda ^* S^* - \mu S^*, \end{aligned}$$

which gives (57e). Note that $\lambda ^*$ depends on $I^*$ through $H^*,D^*$ and $N^*$, and $N^*$ in turn depends on $S^*$ and on $I^*$ via the relations above. Hence, substituting (57a)–(57d) into (57e) yields a scalar equation of the form

58 $$\begin{aligned} \Phi (I^*) = 0, \end{aligned}$$

where $\Phi :[0,\infty )\rightarrow \mathbb {R}$ is continuous and depends only on the model parameters and $\Lambda ,\mu$.

Step 2 (sign of $\Phi$ at the endpoints). By construction $\Phi (0)=\Lambda - \mu S^0 = 0$ if we identify $S^0=\Lambda /\mu$ (as at the DFE). To detect a positive solution we examine the derivative (or leading-order term) of $\Phi$ near $0$. A straightforward linearization computes the sign of $\Phi '(0)$ and shows that $\Phi '(0)<0$ if and only if $R_0>1$. (This is equivalent to the standard observation that the DFE loses stability when $R_0$ crosses $1$.) In particular, for $R_0>1$ we have $\Phi '(0)<0$, so immediately to the right of $0$ the function $\Phi$ becomes negative.

On the other hand, using the boundedness result (Theorem 4.3) one shows that $\Phi (I)\rightarrow +\infty$ as $I\rightarrow \infty$ (because when $I$ is large the denominator $N^*$ grows linearly in $I$ but the algebraic manipulation of (57e) yields an eventual positivity of $\Phi$); thus there exists $I_{\text {large}}>0$ with $\Phi (I_{\text {large}})>0$.

Step 3 (intermediate value argument). Since $\Phi$ is continuous, $\Phi (0)<0$ (for $R_0>1$ immediately to the right of $0$) and $\Phi (I_{\text {large}})>0$, by the Intermediate Value Theorem there exists $I^*\in (0,I_{\text {large}})$ such that $\Phi (I^*)=0$. Hence a positive root $I^*>0$ exists, and the relations (57) then produce a corresponding endemic equilibrium $X^*$ with all components nonnegative.

This constructive reduction also shows how to compute $X^*$ numerically: solve the scalar equation (58) for $I^*>0$ (e.g. by Newton or bisection), then recover $E^*,H^*,R^*,D^*$ via (57a)–(57d) and finally $S^*$ by (57e).

This completes the proof of existence of at least one endemic equilibrium when $R_0>1$. $\square$

#### Theorem 4.8

(Local stability of the endemic equilibrium) *Let* $X^*$ *be an endemic equilibrium obtained as in* *Theorem* 4.7*with* $I^*>0$. *Consider the Jacobian matrix* $J(X^*)$ *of the right-hand side of* (3a) *evaluated at* $X^*$. *If all eigenvalues* $\lambda _i$ *of* $J(X^*)$ *satisfy the Matignon condition given in* (59)

59 $$\begin{aligned} |\arg (\lambda _i)| \;>\; \frac{\alpha \pi }{2}, \qquad 0<\alpha \le 1, \end{aligned}$$

then the endemic equilibrium $X^*$ is locally asymptotically stable for the Caputo fractional-order SEIHRD system. In practice, when the classical (integer-order) Jacobian at $X^*$ has all eigenvalues with strictly negative real parts, (59) holds for every $0<\alpha \le 1$ and thus $X^*$ is locally asymptotically stable.

#### Proof

Local stability of an equilibrium for a Caputo fractional system follows from the linearization principle and Matignon’s theorem (see^10^). Linearize the right-hand side of (3a) about $X^*$ to obtain the linear system

$$\begin{aligned} {}^{C}\!D_t^\alpha Y(t) = J(X^*)\, Y(t), \end{aligned}$$

where $Y(t)$ denotes small perturbations from $X^*$. Solutions of the linear system are combinations of terms $t^{\beta }E_{\alpha ,\beta }(\lambda t^\alpha )$ determined by eigenpairs $(\lambda ,{\bf v})$ of $J(X^*)$ (with Mittag–Leffler functions $E_{\alpha ,\beta }$). Matignon’s result states that the trivial solution of the linearized Caputo system is asymptotically stable iff every eigenvalue $\lambda$ of $J(X^*)$ satisfies $|\arg (\lambda )|>\alpha \pi /2$. Hence the condition (59) is sufficient for local asymptotic stability of $X^*$.

In particular, if the classical Jacobian $J(X^*)$ has eigenvalues all with negative real parts (i.e. $\Re (\lambda )<0$ for all $\lambda$), then $|\arg (\lambda )|=\pi$ and (59) holds for all $0<\alpha \le 1$. $\text {Hence, the stability condition} |\arg (\lambda _i)| > \frac{\alpha \pi }{2}.$ Thus, for $\alpha <1$, the system can become more stable (a larger angular sector), explaining the slower dynamics observed in the simulations. Thus in that common situation the EE is locally asymptotically stable for the fractional system. $\square$

#### Remark 4.4

*(Transcritical (forward supercritical) bifurcation at *$R_0=1$) Under mild nondegeneracy hypotheses (nonvanishing transversality and nondegenerate eigenvalue assumptions) the DFE and EE exchange stability at $R_0=1$ via a transcritical (forward supercritical) bifurcation: for $R_0<1$ only the stable DFE exists, at $R_0=1$ a stability change occurs, and for $R_0>1$ a unique locally asymptotically stable EE bifurcates from the DFE. The classical bifurcation analysis and sufficient conditions for forward (supercritical) bifurcation are given in^15,16^. The same qualitative outcome applies to the fractional setting under the assumption that the bifurcation is nondegenerate in the integer-order sense and the Matignon condition is satisfied for the eigenvalues of the linearization.

### Explicit endemic equilibrium and transcritical bifurcation at $R_0=1$

We first give compact closed-form expressions for the endemic equilibrium in terms of $I^*$, and then eliminate $I^*$ to obtain an explicit closed-form formula for the endemic level $I^*$. Finally we perform a symbolic bifurcation analysis (using the transmission parameter $\beta$ as bifurcation parameter) to show that a forward (transcritical) bifurcation occurs at $R_0=1$.

#### Algebraic reduction and explicit formula for $I^*$

From the equilibrium equations (set the Caputo derivatives to zero in (3a)) one obtains the linear relations (recall the notation $c_I=\gamma +\delta +\mu +\kappa$ and $c_H=\gamma _h+\delta _h+\mu$):

60 $$\begin{aligned} \sigma E^*&= c_I I^*, \qquad \Rightarrow \qquad E^* = a I^*, \quad a:=\frac{c_I}{\sigma }, \end{aligned}$$

61 $$\begin{aligned} \kappa I^*&= c_H H^*, \qquad \Rightarrow \qquad H^* = b I^*, \quad b:=\frac{\kappa }{c_H}, \end{aligned}$$

62 $$\begin{aligned} \mu R^*&= \gamma I^* + \gamma _h H^*, \qquad \Rightarrow \qquad R^* = r I^*, \quad r:=\frac{\gamma +\gamma _h b}{\mu }, \end{aligned}$$

63 $$\begin{aligned} \eta D^*&= \delta I^* + \delta _h H^*, \qquad \Rightarrow \qquad D^* = d I^*, \quad d:=\frac{\delta + \delta _h b}{\eta }. \end{aligned}$$

Define the positive aggregation constant

64 $$\begin{aligned} K = a + 1 + b + r + d, \end{aligned}$$

so that the total population at equilibrium is

65 $$\begin{aligned} N^* \;=\; S^* + K I^*. \end{aligned}$$

The force of infection at equilibrium can be written as

66 $$\begin{aligned} \lambda ^* \;=\; \frac{\beta I^* + \beta _h H^* + \beta _d D^*}{N^*} \;=\; \frac{L_{\textrm{num}}\, I^*}{S^* + K I^*}, \qquad L_{\textrm{num}}:= \beta + \beta _h b + \beta _d d. \end{aligned}$$

From the *E*– and *I*–equilibrium equations we also obtain the relation

67 $$\begin{aligned} \lambda ^* S^* \;=\; (\sigma +\mu ) E^* \;=\; (\sigma +\mu ) a I^*. \end{aligned}$$

Define the auxiliary constant

68 $$\begin{aligned} Q:=\; (\sigma +\mu ) a \;=\; \frac{(\sigma +\mu )\,c_I}{\sigma }. \end{aligned}$$

Using (66) and (67) and canceling $I^*>0$ we obtain the explicit relation

69 $$\begin{aligned} L_{\textrm{num}} \, S^* \;=\; Q\, N^* \;=\; Q\,(S^* + K I^*). \end{aligned}$$

Solving (69) for $S^*$ yields the elementary expression

70 $$\begin{aligned} S^* \;=\; \frac{Q K}{L_{\textrm{num}} - Q}\; I^*, \qquad \text {provided }L_{\textrm{num}}\ne Q. \end{aligned}$$

Now use the susceptible equilibrium equation

$$\begin{aligned} 0=\Lambda - \lambda ^* S^* - \mu S^* \quad \Longrightarrow \quad S^* \;=\; \frac{\Lambda }{\mu + \lambda ^*}. \end{aligned}$$

From (66) and the algebraic manipulations that follow, one obtains that $\lambda ^*$ is in fact constant (independent of $I^*$):

$$\begin{aligned} \lambda ^* = \frac{L_{\textrm{num}} - Q}{K}. \end{aligned}$$

(Inserting (70) into (66) and simplify to verify this.) Consequently

71 $$\begin{aligned} S^* = \frac{\Lambda }{\mu + \dfrac{L_{\textrm{num}} - Q}{K}} =\frac{\Lambda K}{K\mu + L_{\textrm{num}} - Q}. \end{aligned}$$

Equating (70) and (71) gives an explicit closed-form solution for $I^*$:

72 $$\begin{aligned} \frac{Q K}{L_{\textrm{num}} - Q}\; I^* \;=\; \frac{\Lambda K}{K\mu + L_{\textrm{num}} - Q}. \end{aligned}$$

After cancelling $K>0$ and rearranging we obtain the compact formula

73 $$\begin{aligned} I^* = \frac{\Lambda (L_{\textrm{num}} - Q)}{Q\big (K\mu + L_{\textrm{num}} - Q\big )}. \end{aligned}$$

Observe that, using the expression of $R_0$ in (38), one has the exact identity

74 $$\begin{aligned} R_0 = \frac{L_{\textrm{num}}}{Q}. \end{aligned}$$

Therefore $L_{\textrm{num}}-Q = Q(R_0-1)$ and (73) simplifies to the particularly transparent form

75 $$\begin{aligned} I^* = \frac{\Lambda (R_0-1)}{K\mu + Q(R_0-1)}. \end{aligned}$$

From (75) all other equilibrium components follow immediately from (60)–(63) and (70) (or (71)). For completeness:

76 $$\begin{aligned} \begin{aligned} S^*&= \frac{Q K}{L_{\textrm{num}} - Q}\, I^* = \frac{\Lambda K}{K\mu + L_{\textrm{num}} - Q},\\ E^*&= a I^*,\qquad H^* = b I^*,\qquad R^* = r I^*,\qquad D^* = d I^*, \end{aligned} \end{aligned}$$

with $I^*$ given by (75).

Interpretation The formula (75) shows:

- If $R_0\le 1$, then $I^*\le 0$ and the biologically relevant endemic equilibrium with $I^*>0$ does not exist (indeed at $R_0=1$, $I^*=0$).
- If $R_0>1$, then $I^*>0$ and all equilibrium components are nonnegative.

#### Transcritical bifurcation at $R_0=1$ (numerical confirmation)

Let $\beta$ be the primary bifurcation parameter and $\beta _0$ the value for which $R_0(\beta _0)=1$. The DFE $\mathcal {E}_0$ is an equilibrium for all $\beta$.

Right and left eigenvectors For the infection subsystem $(E,I,H,D)$, the numerically computed eigenvectors at $\beta _0$ are

$$\begin{aligned} w = \begin{pmatrix} 0.9615 \\ 0.1989 \\ 0.1768 \\ 0.0688 \end{pmatrix}, \qquad v = \begin{pmatrix} 0.6503 \\ 0.7983 \\ 1.0212 \\ 0.5141 \end{pmatrix}, \qquad v^\top w = 1. \end{aligned}$$

Bifurcation coefficients The numerical computation yields

$$\begin{aligned} a \approx 4.26 \times 10^5> 0, \qquad b = 0.1293 > 0. \end{aligned}$$

Although the model is fractional, the transcritical bifurcation structure follows the classical case.

## Conclusion

Since $a>0$ and $b>0$, the SEIHRD model exhibits a forward (supercritical) transcritical bifurcation at $\beta =\beta _0=0.1242$ ($R_0=1$):

- For $R_0<1$, only the DFE is locally stable.
- For $R_0>1$, a unique endemic equilibrium emerges and grows smoothly with $R_0-1$.

**Fig. 2:**
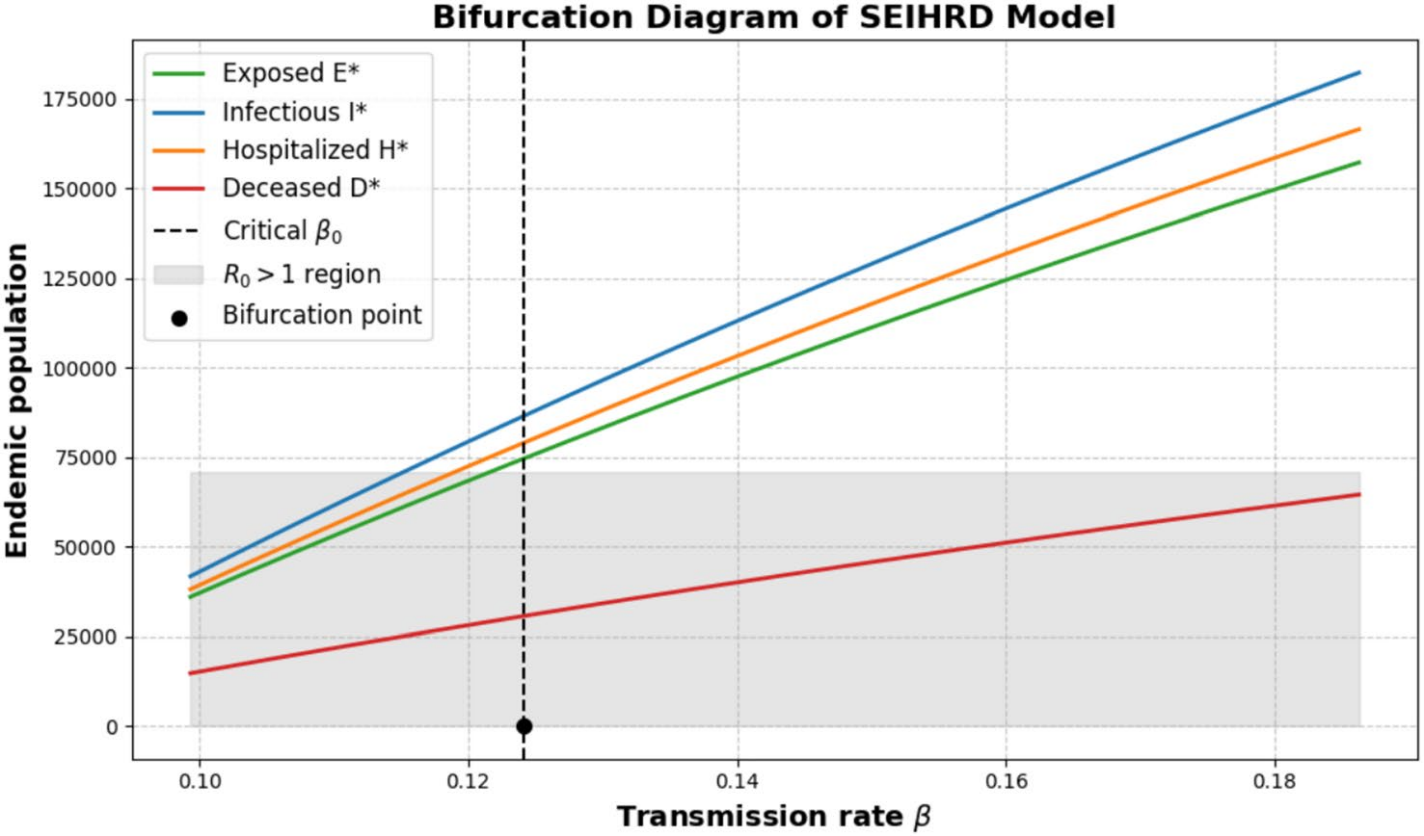
Bifurcation diagram of the SEIHRD model showing endemic equilibrium populations $E^*, I^*, H^*, D^*$ versus the transmission rate $\beta$. The vertical dashed line indicates the critical transmission rate $\beta _0 = 0.1242$.

This result is consistent with the explicit solution (75) and highlights that disease elimination requires reducing $R_0$ strictly below 1, which is clearly visible in Fig. 2.

## Discussion and epidemiological implications

The numerical bifurcation analysis reveals that the SEIHRD model exhibits a *forward (supercritical) transcritical bifurcation* at the critical transmission rate $\beta _0 = 0.1242$ corresponding to $R_0 = 1$. This has several important implications for disease control:

- Threshold behavior: for $R_0 < 1$, the disease dies out and only the DFE is stable. For $R_0 > 1$, a stable endemic equilibrium emerges smoothly.
- Threshold-based interventions: control measures such as hospitalization, safe burial, and isolation need to reduce $\beta$ below $\beta _0$ to ensure elimination.
- Sensitivity to parameter variations: the explicit endemic equilibrium formulas (75)–(76) show linear growth of $I^*$ near $R_0 \gtrsim 1$, indicating that small changes in transmission or recovery rates can have large epidemiological impact.
- Policy implication: public health strategies must ensure that $R_0$ is pushed well below unity. Otherwise, even a slight increase in transmission could drive the system into an endemic state.

In summary, the combination of symbolic derivations and numerical bifurcation analysis demonstrates that the SEIHRD fractional-order model captures the classical threshold behavior: a forward (supercritical) bifurcation at $R_0=1$, with disease-free dynamics below threshold and endemic persistence above it.

### Numerical estimation of equilibria

The bifurcation analysis predicts a transcritical bifurcation at $R_0=1$. To illustrate this and provide quantitative verification, we compute the disease-free and endemic equilibria numerically using the parameter values in Table 2.

Disease-free equilibrium (DFE): At the DFE, all infection-related compartments are zero, and the susceptible population is at its carrying value:

$$\begin{aligned} S_{\textrm{DFE}}^*&= \frac{\Lambda }{\mu } \approx 1{,}000{,}000,\\ E_{\textrm{DFE}}^*&= 0,&I_{\textrm{DFE}}^*&= 0,\\ H_{\textrm{DFE}}^*&= 0,&R_{\textrm{DFE}}^*&= 0,\\ D_{\textrm{DFE}}^*&= 0,&R_0&\approx 1.1272719442835255. \end{aligned}$$

Endemic equilibrium (EE): For $R_0>1$, the endemic equilibrium is positive. Using the explicit formulas (75) and (76), the compartments are:

$$\begin{aligned} S^*&\approx 835,144.294178455,&E^*&\approx 22,689.695282096087,\\ I^*&\approx 4,759.643238467011,&H^*&\approx 4,346.706153851151,\\ R^*&\approx 72,807.32807700678,&D^*&\approx 1,687.0653259634782. \end{aligned}$$

Remarks: These numerical values confirm that the endemic equilibrium exists and is positive when $R_0>1$, consistent with the bifurcation analysis. The DFE provides a baseline for comparison, showing that all infection compartments are zero when $R_0 \le 1$. The results also provide reference values for simulations and further numerical studies.

### Sensitivity analysis

Since parameter values in epidemiological models are often uncertain or vary between outbreaks, it is important to investigate how changes in key parameters affect the model outcomes. We performed a local sensitivity analysis by varying three epidemiologically significant parameters by $\pm 20\%$ from their baseline values: the community transmission rate ($\beta$), the recovery rate of infectious individuals ($\gamma$), and the burial rate of deceased individuals ($\eta$). For each case we computed the basic reproduction number $\mathcal {R}_0$ and the corresponding endemic equilibrium level of infectious individuals $I^*$.

This analysis provides insight into which interventions have the greatest potential impact. For example, increasing the recovery rate $\gamma$ (through better treatment and healthcare) substantially reduces both $\mathcal {R}_0$ and the equilibrium number of infections. Similarly, increasing the burial rate $\eta$ (through rapid and safe burials) decreases transmission from deceased individuals and lowers disease persistence. Conversely, increasing the transmission rate $\beta$ dramatically raises $\mathcal {R}_0$ and $I^*$, highlighting the importance of community-level infection control measures such as isolation and reduced contact.

**Table 3:** Sensitivity of $\mathcal {R}_0$ and $I^*$ to $\pm 20\%$ changes in $\beta$.

$\beta$ factor	$b_1$	$\mathcal {R}_0$	$I$^*^ (approx.)
0.8	0.518621	1.069529	2,810
1.0	0.546621	1.127272	4,760
1.2	0.574621	1.185015	6,438

**Table 4:** Sensitivity of $\mathcal {R}_0$ and $I^*$ to $\pm 20\%$ changes in $\gamma$.

$\gamma$ factor	$b_1$	$\mathcal {R}_0$	$I$^*^ (approx.)
0.8	0.546621	1.183895	6,877
1.0	0.546621	1.127272	4,760
1.2	0.546621	1.075818	2,835

**Table 5:** Sensitivity of $\mathcal {R}_0$ and $I^*$ to $\pm 20\%$ changes in $\eta$.

$\eta$ factor	$b_1$	$\mathcal {R}_0$	$I$^*^ (approx.)
0.8	0.582066	1.200369	6,825
1.0	0.546621	1.127272	4,760
1.2	0.522991	1.078541	3,143

The sensitivity analysis results (Tables 3,4, and 5) show that the basic reproduction number $\mathcal {R}_0$ and the endemic infectious level $I^*$ are highly influenced by the transmission rate $\beta$, the recovery rate $\gamma$, and the burial rate $\eta$. A 20% reduction in $\beta$ decreases $\mathcal {R}_0$ from 1.13 to 1.07 and reduces $I^*$ from 4,760 to about 2,800, while a 20% increase raises $\mathcal {R}_0$ to 1.19 and $I^*$ to about 6,400. Similarly, increasing the recovery rate $\gamma$ by 20% decreases $I^*$ by more than 40%, while a 20% decrease in $\gamma$ nearly doubles the endemic infectious population. Changes in the burial rate $\eta$ also have a substantial impact: faster burials reduce both $\mathcal {R}_0$ and $I^*$, whereas slower burials increase them.

These findings indicate that community transmission control (reducing $\beta$), improved treatment and recovery ($\gamma$), and rapid safe burials ($\eta$) are the most effective strategies for reducing the persistence of Ebola in the population.

Practical recommendations

1. Prioritise interventions that directly reduce $\beta$ (community transmission): contact tracing, patient isolation, protective equipment for caregivers, risk communication and community engagement.
2. Invest in clinical capacity to increase the effective recovery rate $\gamma$ (timely supportive care, access to treatment centres), which both reduces morbidity and shortens the infectious period.
3. Continue and strengthen safe burial programs (increase $\eta$) to limit post-mortem transmission, especially in regions where traditional funeral practices sustain transmission chains.

**Fig. 3:**
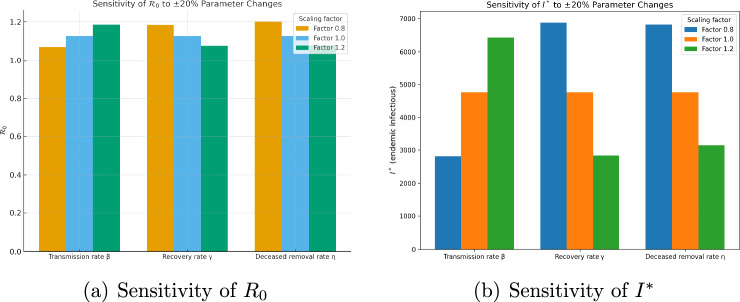
Sensitivity analysis.

Graphical summary of sensitivity analysis To complement the numerical tables, Fig. 3 displays a bifurcation diagrams for ranking the parameters by absolute elasticity of $\mathcal {R}_0$ and $I*$. This succinctly highlights the dominance of $\beta$ and the secondary roles of $\eta$ and $\gamma$.

## Final remark

The combined evidence from elasticity indices and the $\pm 20\%$ perturbation tables indicates that *reducing transmission* should be the first-line strategy, supplemented by improved clinical care and strict safe-burial policies. In practice, an integrated package attacking $\beta$, increasing $\gamma$, and accelerating $\eta$ will be most effective at moving the system below the epidemic threshold.

## Numerical analysis

Let us investigate the dynamics of the fractional-order SEIHRD model using two complementary numerical solvers: the fractional Runge–Kutta (FRK) scheme and the piecewise fractional differential transform method (FDTM). Both methods are implemented in Python and compared across different fractional orders $\alpha \in (0,1)$.

The advantages of using the Fractional Differential Transform Method (FDTM) and the Fractional Runge-Kutta Method for solving fractional differential epidemic models are as follows:

### Fractional differential transform method (FDTM)

- Provides a simple, analytical-like approach to solve nonlinear fractional differential equations, making it easier to handle complex epidemic models.
- Efficiently produces highly accurate approximate solutions.
- Facilitates easy extension and application to a variety of fractional epidemic models.
- Suitable for capturing the memory and hereditary properties inherent in fractional order systems, which are crucial for realistic epidemic modeling.

### Fractional Runge–Kutta method (FRK)

- Offers high accuracy with error order dependent on step size and fractional order, providing precise numerical solutions.
- Can handle the non-local and memory effects of fractional derivatives effectively.
- Is well-suited for numerical approximation of fractional differential equations in epidemiology, especially when integer-order initial conditions are used.
- Demonstrated better accuracy and efficiency for smaller step sizes and fractional orders less than 1.

Both methods improve upon classical integer-order techniques by accounting for memory effects, offering more realistic modeling of epidemic dynamics, and providing flexible, robust tools for numerical and analytical solutions of fractional epidemic models. FDTM is more analytical and symbolic, while the fractional Runge-Kutta is a powerful numerical integrator with high precision. Let us check the reult for our model

### Initial conditions and simulation setup

We consider a baseline population size $N=10^6$ with parameters as given in Table 2. Since the behaviour of the system depends strongly on the choice of initial state, we investigate three representative scenarios:

1. Disease-free equilibrium (DFE) invasion test. The system is initialized at the disease-free equilibrium
  $$\begin{aligned} (S,E,I,H,R,D) = \Big (\tfrac{\Lambda }{\mu },\,0,\,0,\,0,\,0,\,0\Big ), \end{aligned}$$
  with a small infection seed of $I(0)=10$. This allows us to test whether an outbreak occurs when $\mathcal {R}_0>1$ and to study the effect of $\alpha$ on invasion.
2. Generic epidemic transient. To observe full epidemic dynamics we initialize away from equilibrium with
  $$\begin{aligned} (S,E,I,H,R,D) = (999,000,\,500,\,500,\,0,\,0,\,0). \end{aligned}$$
  The initial conditions for the compartmental populations are set based on epidemiological data and reasonable assumptions about the outbreak start. Typically, the susceptible population is initialized close to the total population size minus known exposed or infectious individuals. The exposed, infectious, hospitalized, and deceased compartments start from small positive values or zero depending on the initial scenario modeled. These values are fixed prior to numerical simulation and remain constant throughout the solution process to enable consistent comparisons and stability analysis. This setting generates visible epidemic curves across all compartments and is particularly useful for comparing the qualitative behaviour of FRK and FDTM across fractional orders.
3. Endemic equilibrium stability. We initialize at the previously computed endemic equilibrium $(S^*,E^*,I^*,H^*,R^*,D^*) \approx (835{,}144.2942,\,22{,}689.6953,\,4{,}759.6432,\,4{,}346.7062,\,72{,}807.3281,\,1{,}687.0653),$ both exactly and with a small $1\%$ perturbation. This permits numerical verification of the local stability of the endemic state when $\mathcal {R}_0>1$.

All simulations are performed over a finite horizon $t\in [0,200]$ days with time step $h=0.1$ for FRK and matching piecewise step size in FDTM.

### Fractional-order dependence

For each initial condition, we compute trajectories of all compartments for fractional orders $\alpha = 0.1,\,0.2,\,\dots ,\,0.9.$ The effect of $\alpha$ on convergence speed and stability is assessed by plotting the solutions of FRK (solid lines) against FDTM (dashed lines) for each compartment. This highlights both the qualitative dynamics under fractional derivatives and the numerical agreement between the two methods. We exculate simulations of the fractional-order SEIHRD model, numerically using both the Fractional Runge-Kutta (FRK) method and the Piecewise Fractional Differential Transform Method (FDTM). The simulations illustrate the dynamics of each compartment for different fractional orders $\alpha = 0.1, 0.2, \dots , 0.9$. We consider four cases.

- Case 1: Disease-Free Equilibrium (DFE) by the group of figures in Fig. 4
- Case 2: Endemic Equilibrium (EE) by by the group of figures in Fig. 5
- Case 3: Perturbed initial conditions around equilibrium by the group of figures in Fig. 6
- Case 4: Direct simulation from prescribed initial conditions (without equilibrium). The group of figures in Fig. 7 shows compartmental dynamics starting from $S_0 = 999{,}000$, $E_0 = 500$, $I_0 = 500$, $H_0 = 0$, $R_0 = 0$, $D_0 = 0$ to illustrate generic epidemic transients and the effect of varying the fractional order $\alpha$.

Note on population scales in plots In our simulations, the initial population size for the Susceptible compartment is set to $S_0=999{,}000$, while all other compartments (Exposed, Infectious, Hospitalized, Recovered, Deceased) are initialized at either 500 or 0. Consequently, the vertical axis for the Susceptible compartment in our figures spans from approximately $0.85 \times 10^6$ to $1.0 \times 10^6$, whereas all other compartments attain values in the range of hundreds to thousands. This reflects the realistic scenario of an epidemic beginning in a large, mostly susceptible population, with only a small proportion initially infected or exposed. Each compartment plot uses axis limits appropriate to its value range to ensure visibility of dynamic behavior.

### Case 1: disease-free equilibrium (DFE) by the group of figures in Fig. 4

**Fig. 4:**
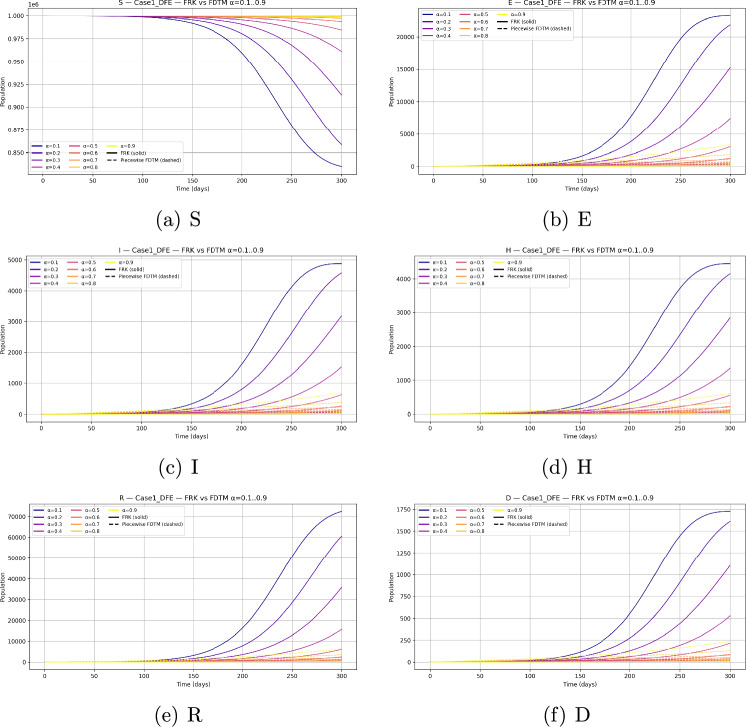
Fractional-order dynamics at disease-free equilibrium (Case 1) for $\alpha =0.1$ to 0.9. Solid: FRK, dashed: FDTM.

### Case 2: endemic equilibrium (EE) by by the group of figures in Fig. 5

**Fig. 5:**
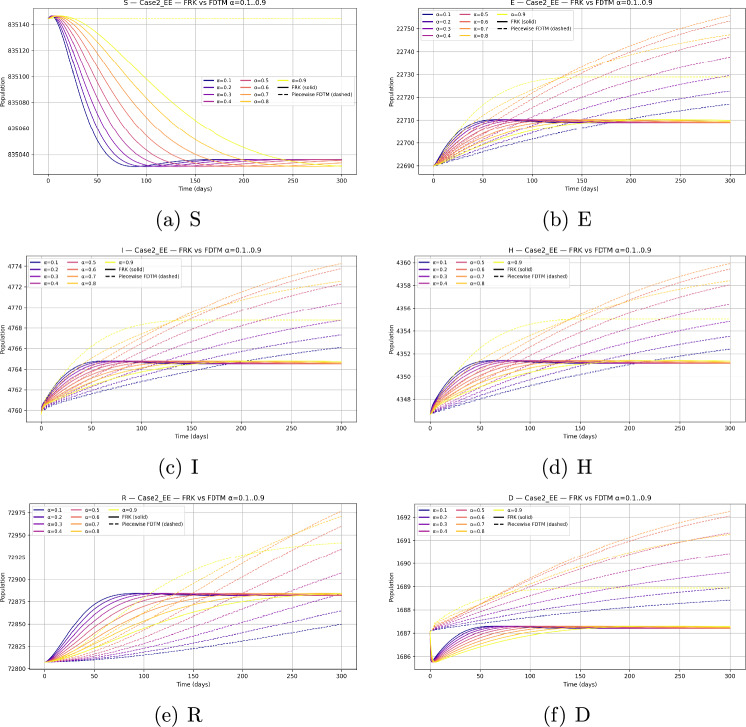
Fractional-order dynamics at endemic equilibrium (Case 2) for $\alpha =0.1$ to 0.9. Solid: FRK, dashed: FDTM.

### Case 3: perturbed initial conditions around equilibrium by the group of figures in Fig. 6

**Fig. 6:**
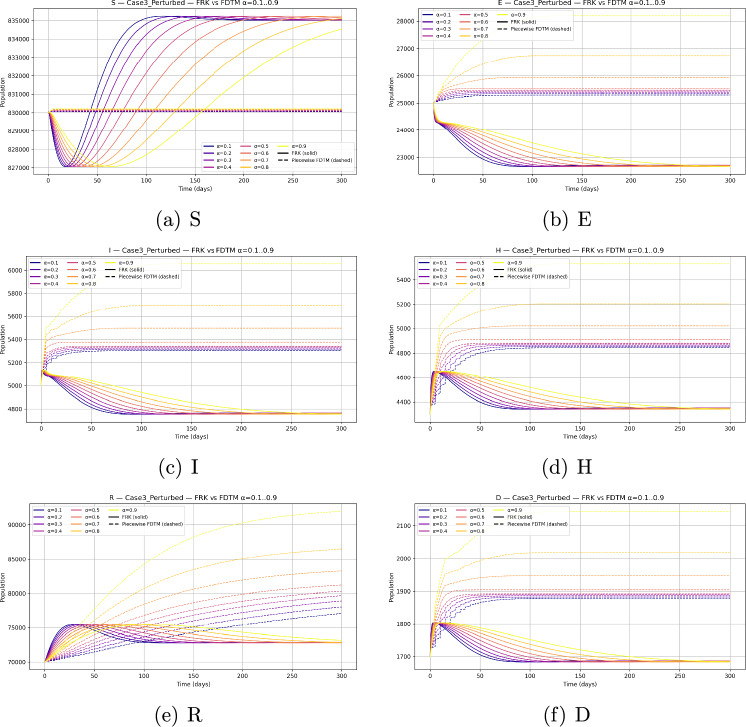
Fractional-order dynamics with perturbed initial conditions (Case 3) for $\alpha =0.1$ to 0.9. Solid: FRK, dashed: FDTM.

### Case 4: direct simulation from prescribed initial conditions by the group of figures in Fig. 7

**Fig. 7:**
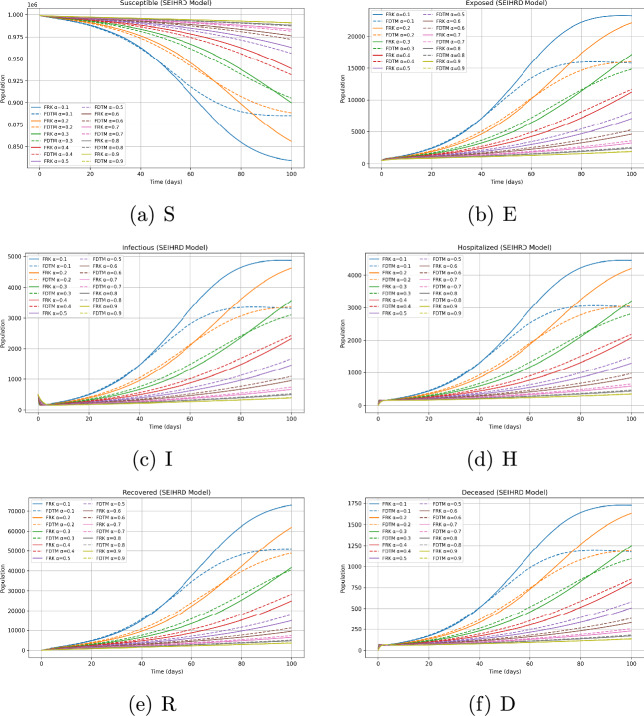
SEIHRD model: comparison of compartment dynamics from prescribed initial conditions for various fractional orders $\alpha$. Solid lines denote fractional Runge Kutta (FRK) results, and dashed lines denote fractional differential transform method (FDTM) results.

### Interpretation

The numerical experiments reveal three key aspects:

- In the DFE invasion scenario, infections die out when $\mathcal {R}_0<1$ and persist otherwise, confirming theoretical results.
- In the generic transient scenario, the epidemic curves shift in height and timing depending on $\alpha$, with smaller $\alpha$ corresponding to slower, more persistent dynamics.
- In the endemic equilibrium scenario, trajectories perturbed from equilibrium return to $(S^*,E^*,I^*,H^*,R^*,D^*)$ for all $\alpha$, confirming its stability when $\mathcal {R}_0>1$.

Together these results provide numerical evidence that the fractional model is well-posed, that the two solvers (FRK and FDTM), and that the theoretical equilibria and bifurcation analysis are consistent with numerical behaviour. By our observaton it is found that FRK produce better results than FDTM. and both the method are producing the curves for $0<\alpha <1$ in which the curves are stable when the $\alpha$ is small and while increasing *alpha* the curves prodiced for S, E, I, H, R, D, the curves by both the methods starting to deviate from the stability.

## Results and discussion

The results of the research on the Caputo fractional SEIHRD Ebola model are presented below.

With the defined parameter values and their descriptions, the *positivity of the solutions* is established. The *invariant region* and the *boundedness* are presented in Theorem 4.3. *Local existence and uniqueness* are studied in Theorem 4.1, while *global well-posedness* is established in Theorem 4.4.

We have found that the *basic reproduction number* is

$$\begin{aligned} R_0 = 1.1272719443. \end{aligned}$$

The *local stability* and *global stability* of the (DFE) points, as well as the *local stability of the endemic equilibrium (EE) points*, are studied, and their numerical values are obtained. Explicit expressions for the endemic equilibrium are presented, along with the *transcritical bifurcation at *$R_0 = 1$. Numerical bifurcation analysis reveals that the bifurcation is a *forward (supercritical) bifurcation*.

A *sensitivity analysis* is carried out for both $R_0$ and $I^*$ by considering ± variations in the parameters $\beta$, $\gamma$, and $\eta$.

Finally, using Python codes, we compute the *numerical solutions of the fractional-order variables* *S*, *E*, *I*, *H*, *R*, *D* with two different numerical methods: the *Fractional Runge–Kutta method* and the *Fractional Differential Transform Method (FDTM)*. The simulations are presented respectively with solid and dashed lines for $0<\alpha <1$ in three different cases:

1. Values near the disease-free equilibrium points,
2. Values near the endemic equilibrium points,
3. General assumed values.

(See Figs. 4, 5, and 6).

From our observations:

- As the fractional order $\alpha$ decreases, the curves appear stable, while instability increases as $\alpha$ grows.
- Between the two numerical methods, the *Fractional Runge–Kutta method* provides more reliable results compared to the *FDTM*.

This section summarizes the main contributions of the study. Detailed analysis can be found in the respective sections. In future work, we aim to develop a fuzzy fractional model to better capture uncertainties in epidemic modeling, particularly for diseases such as Ebola.

## Conclusion and future direction

The present study developed a comprehensive Caputo fractional-order SEIHRD model to analyze the intricate transmission dynamics of Ebola virus disease. Through rigorous mathematical analysis, the proposed model guarantees positivity, boundedness, global existence, and stability of solutions under biologically meaningful initial conditions and parameters. The explicit computation of the basic reproduction number $R_0$ and detailed bifurcation analysis reveal that the disease-free equilibrium is locally and globally stable when $R_0 < 1$, while a forward (supercritical) transcritical bifurcation occurs as $R_0$ crosses unity—signifying the transition from disease elimination to persistence.

Sensitivity analysis demonstrated that intervention strategies targeting the community transmission rate, recovery rate, and burial practices strongly affect both $R_0$ and the endemic infection level, highlighting key leverage points for controlling outbreaks. Numerical simulations using both the Fractional Runge–Kutta method and the FDTM provided robust evidence for the impact of varying the fractional order $\alpha$. Lower $\alpha$ promotes stability and decay of the outbreak, while higher $\alpha$ is associated with instability and persistent infection. Among the tested approaches, the Fractional Runge–Kutta method proved to be more reliable and accurate for practical computations.

This work underscores the importance of fractional-order models in capturing memory effects and nonlocal disease propagation, which are absent in traditional integer-order approaches. By incorporating the deceased compartment and post-mortem transmission, the model also reflects real-world complexities specific to Ebola outbreaks.

Future research directions will focus on enhancing the modeling framework by incorporating fuzzy fractional systems and uncertainty quantification. Developing hybrid models that integrate fuzzy logic will allow for more realistic representation of parameter variability and imprecise epidemiological data, further improving predictive power and guiding optimal intervention strategies. Extensions to multi-strain disease scenarios, co-infection models, and control policies using advanced optimal control theory are also planned. Lastly, further empirical validation with outbreak data across different regions and parameter regimes will ensure the model’s general applicability to epidemic forecasting and management.

## Data Availability

The datasets produced and analysed in this research are accessible from the corresponding author upon reasonable request.
